# Molecular Characterization of Extracellular Vesicles From Human B Cell Lymphomas: Methodological Comparison to Vesicles From Patient Serum

**DOI:** 10.1002/jex2.70107

**Published:** 2026-02-22

**Authors:** Md Nasir Uddin Badal, Tiia Koivula, Md Khirul Islam, Laura Lehtinen, Otto Kauko, Janne Leivo, Ilkka Heinonen, Saara Hämälistö

**Affiliations:** ^1^ Institute of Biomedicine University of Turku and FICAN WEST Turku Finland; ^2^ Turku PET Centre University of Turku, Åbo Akademi University, and Turku University Hospital Turku Finland; ^3^ Department of Life Technologies and InFLAMES Research Flagship Center, Division of Biotechnology University of Turku Turku Finland; ^4^ Turku Bioscience Centre University of Turku and Åbo Akademi University Turku Finland; ^5^ The UKK Institute for Health Promotion Research Tampere Finland

**Keywords:** B cell lymphoma subtypes, extracellular vesicles, immunoassay, MS analysis, patient serum, tetraspanins

## Abstract

Human B cell lymphomas represent a clinically heterogeneous disease group with lack of liquid biomarkers for specific subtype classification. Extracellular vesicles (EVs) hold promises as non‐invasive biomarkers, yet their subtype‐specific characteristics and clinical utility in these diseases remain largely underexplored. In this study, we have investigated the basic molecular and physical features of EVs from diffuse large B cell lymphoma (DLBCL) cells and from lymphoma patients’ serum. Data from e.g., electron microscopy (EM), Western blotting (WB), immunoassay and mass spectrometry (MS) revealed that the two main DLBCL cell subtype EVs differ in protein expression profile and in overall EV size, with the ABC (Activated B Cell) type having smaller EVs than the GCB (Germinal Centre B cell) type. The ABC type EVs were found significantly more enriched with tetraspanins CD81 and CD9. Parallel experimentation on lymphoma serum EVs revealed shared markers with lymphoma cell line EVs, and that B cell specific marker CD19 can be detected among other serum EVs. Also, to successfully detect markers, e.g. Hsp70 or CD44, in serum EVs we demonstrated to require more intense sample preparation in specific assays. While more patient studies are needed in the future, this pilot study paves the way for understanding the molecular differences in the DLBCL subtypes and for detecting them in the lymphoma EVs.

## Graphical abstract

This figure summarises the main steps in this study, including used methodology. The extracellular vesicles were isolated from human B lymphoma subtype cells and serum of patients by SEC and characterized for EV sizes and marker expression e.g. tetraspanin enrichment in the EVs. Key findings demonstrate differences in EV sizes and marker expression in the distinct lymphoma subtype EVs. Created in BioRender. Hämälistö, S. (2026) https://BioRender.com/oduv5ok

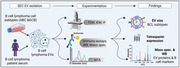

## 1 Introduction

Extracellular vesicles (EVs) are membrane‐bound nanostructures secreted by virtually all cell types into the extracellular space and circulation (Konoshenko et al., 2018). These vesicles act as vital mediators of intercellular communication, transporting a diverse cargo of proteins, lipids, nucleic acids, and other bioactive molecules that reflect the physiological or pathological state of the parent cell (Konoshenko et al., 2018; Oeyen et al., 2018; Srinivasan et al., 2019). Due to this characteristic, EVs have gained attention as promising candidates for diagnostic, prognostic, and therapeutic applications (Kumar et al., 2024; Su et al., 2025; Wang et al., 2025).

EVs comprise several subtypes, including exosomes and microvesicles, which differ in size, biogenesis, and molecular composition (Hartjes et al., 2019). Exosomes originate from endosomal compartments, whereas ectosomes (also referred to as microvesicles) are formed by direct budding from the plasma membrane (Welsh et al., 2024). The concentration of EVs, particularly exosomes, is notably high in biological fluids such as blood, and their levels can fluctuate depending on disease states (Valadi et al., 2007). Despite their small size, EVs hold immense potential in diverse fields including oncology (Semeradtova et al., 2025) immunology (Buzas, 2023), and regenerative medicine (Moghassemi et al., 2024), prompting extensive research into their biological functions and clinical utility (Nieuwland et al., 2022).

Among haematological malignancies, B cell lymphomas represent a heterogeneous group arising from the malignant transformation of B lymphocytes. Diffuse large B cell lymphoma (DLBCL) is the most common subtype, accounting for approximately 40% of newly diagnosed non‐Hodgkin lymphomas (Swerdlow et al., 2016). It is characterized by substantial heterogeneity in morphology, molecular features, clinical behaviour, and response to therapy (Habermann, 2012). Based on gene expression profiling, the DLBCLs are categorized into two major molecular subtypes: germinal centre B cell‐like (GCB) and activated B cell‐like (ABC) (Matthiesen et al., 2022). While DLBCL has traditionally been classified into GCB and ABC subtypes based on cell‐of‐origin, subsequent genomic studies have revealed additional genetic and mutational subtypes such as MCD (mutations in MYD88 and CD79B), BN2 (translocation in BCL6 and mutation in NOTCH2), EZB, (mutations in EZH2 and translocation in BCL2) and many others which influence treatment response and prognosis (Lenz et al., 2008; Schmitz et al., 2018). Current standard methods to genetically classify the DLBCLs include gene expression profiling of frozen tissue samples or the Lymph2Cx assay of paraffined tissues; however, these approaches are impractical for routine clinical or may suffer from limitations such as RNA degradation (Alizadeh et al., 2000; Lenz et al., 2008; Scott et al., 2014).

To overcome these limitations, liquid biopsy has emerged as a minimally invasive technique that enables molecular profiling using circulating biomarkers, including cell‐free DNA, circulating tumour cells, and EVs (Gutiérrez‐García et al., 2011). Among these, EVs are promising due to their continuous presence in circulation and their capacity to mirror tumour‐specific molecular signatures. However, the EVs in the heterogeneous lymphomas and the detection of BCL specific markers in a mixture of EVs in patient derived samples is still understudied but needed for understanding their potential in identifying BCL‐specific EV signature (Aghayan et al., 2025).

The isolation and analysis of EVs are inherently challenging due to their nanoscale size and the complexity of biological matrices such as plasma or serum (De Sousa et al., 2023; Jia et al., 2022; Van Der Pol et al., 2016). These challenges are compounded in DLBCL research, where direct comparisons of EV characterization techniques across serum‐ and cell culture‐derived sources remain limited. Inconsistencies in isolation protocols and analytical targets further hinder reproducibility and the establishment of standardized workflows (Jia et al., 2022; Nieuwland et al., 2022; Wadenpohl et al., 2024).

Studying blood‐derived EVs is essential for clinically translatable findings, while EVs from cell lines remain invaluable for mechanistic studies and biomarker discovery (Moghassemi et al., 2024). Yet, current EV studies vary significantly in their use of characterization techniques such as nanoparticle tracking analysis (NTA), TEM, mass spectrometry (MS), Western blotting (WB), and immunoassays like ELISA resulting in inconsistent data thus complicating cross‐study comparisons (De Sousa et al., 2023; Hartjes et al., 2019; Théry et al., 2018). The expression profiles of distinct molecules e.g. tetraspanins CD9, CD63, and CD81 are utilized to assess EV enrichment, purity, and heterogeneity in different sample types and they may help categorize EV populations (Mizenko et al., 2024; Rydland et al., 2023; Welsh et al., 2024).

Along these lines, this study focuses on the EV characteristics of DLBCL, its 2 main subtypes and lymphoma serum EVs. Moreover, we address the commonalities and differences in sample processing after identical isolation from cell lines and serum. By evaluating key parameters such as EV proteome and the distinct expression of tetraspanins as well as lymphoma‐specific markers (CD19 and CD20) we assess the relative performance and limitations of each analytical technique. In addition, we highlight robust differences observed between the EV samples analysed in this study. Although limited with sample quantity, this methodological investigation provides insights that may support the development of non‐invasive approaches for lymphoma profiling and putatively future patient stratification.

## 2 Methods and Materials

### 2.1 Cell Culture and EV Collection

The DLBCL cell lines used in this study were generous gift by the Sirpa Leppä Laboratory at Helsinki University Hospital, Finland (Table 1). Cells were cultured in RPMI 1640 medium supplemented with 10% foetal bovine serum (FBS) (before experimentation, cultured in exosome‐depleted FBS) and 1% penicillin‐streptomycin. Cultures were maintained in T75 flasks at 37°C with 5% CO_2_ in a humidified incubator. Cell viability was assessed prior to EV isolation, and all cultures demonstrated viability above 95%. Conditioned media were collected when cell confluency reached 80%–90%. To remove cell debris, the collected media were centrifuged at 1500 rpm for 10 minutes. The resulting supernatant was concentrated using centrifugal filters with a 10 kDa molecular weight cutoff (MWCO) by centrifugation at 3900 rpm for 30 minutes, reducing the volume to approximately 150 µL. This was followed by an additional centrifugation step at 15,000 x g for 30 minutes at 4°C to remove residual debris. The EV‐enriched supernatants were either immediately processed for SEC‐based EV isolation or stored at ‐80°C for later use.

**TABLE 1:** Information of the DLBCL cell lines used in the study.

Cell lines	Abbreviation used	Disease	Subtype	Tissue	ATCC/DSMZ
U‐2932	U‐2	DLBCL	ABC	Ascites	ACC 633
Riva	Ri	DLBCL	ABC	N/A	N/A
SUDHL‐4	S‐4	DLBCL	GCB	Peritoneal effusion	CRL‐2957
OCI‐LY7	O‐7	DLBCL	GCB	Peripheral blood	ACC 688

### 2.2 Serum Sample Collection

The work on patient serum was conducted under formal approval and registration as required by the local legislation (Ethical number 72/1801/2018) at Turku University Hospital. The four‐sample cohort included three male and one female patient diagnosed with either DLBCL, Hodgkin or small lymphocytic lymphoma (Table 2). The patients had an average age of 51 years and were at various cancer stages ranging from stage II to stage IV. Venous blood was drawn from patients: 3ml whole blood was collected to EDTA tubes from which 1ml serum was separated and stored at ‐80 Celsius for further analysis.

**TABLE 2:** Information of the patients with various forms of B cell lymphoma.

Patient ID	Disease	Age	Gender	BMI
Pt#01	DLBCL	63	M	27.7
Pt#02	Hodgkin	20	M	27.7
Pt#03	Hodgkin	52	M	26.4
Pt#04	Small lymphatic lymphoma	69	F	27.7

### 2.3 Size Exclusion Chromatography: EV Isolation From Cell Culture and Serum Sample

EVs were isolated from both concentrated cell culture supernatants and serum samples using qEV single Gen2 70 nm SEC columns (see Table 3 for antibodies and reagents). All columns were prepared by flushing with two column volumes (6 mL) of 0.1 µm filtered phosphate‐buffered saline (PBS) to equilibrate the matrix. A total of 150 µL of either concentrated cell culture supernatant or undiluted serum was loaded onto the equilibrated column. Once the sample was fully absorbed, 700 µL of filtered PBS was added to elute the void volume, which was discarded. Subsequently, fractions 1–8 (each 170 µL) were collected, and to ensure a sufficient yield for analysis, every two consecutive fractions were pooled into final four samples F1‐F4 (∼340 µL per pooled fraction). Apart from analyses performed on separate fractions F1‐F4, for downstream analyses, pooled fractions F1‐F3 (1‐6 from SEC) were combined into a single tube, yielding a total volume of ∼1020 µL; sample F4 was not included in down‐stream analyses. The collected EV fractions were then concentrated using Amicon protein concentrator (Merck Millipore) with a 10 kDa molecular MWCO. Protein concentrations in EV preps were measured using a NanoDrop spectrophotometer (A260/A280), and samples were stored at ‐80°C for downstream analyses.

**TABLE 3:** List of antibodies and reagents.

Name	Company	Catalog number	Application
a‐CD63‐AF488 (clone H5C6)	Novus Biologicals	NBP2‐42225AF488	IF
a‐CD81‐560/585	Biotium	#P005‐560	IF
a‐CD19	Cell Signaling	3574S	WB
a‐CD20	Roche	5267099001	WB
a‐Hsp70	Bio‐Rad	MCA6018	WB
a‐CD44	Bio‐Techne	NBP1‐31488	WB
RPMI cell culture media	Thermo‐Fisher Scientific	61870010	Cell culture
Foetal bovine serum (exosome depleted)	Thermo Fisher scientific	A2720802P	SEC EV isolation
qEV Single Gen 2 70 nm column	Izon	IC1‐70	SEC EV isolation
ApoA1	Proteintech	14427‐1‐AP	WB
GAPDH	Santa Cruz	sc‐47724	WB
Mouse CD63 antibody	Invitrogen	DAQ‐PF150916T1	WB, iEM
Mouse CD9 antibody	Proteintech ab	MAB1880	WB iEM, IF
Mouse CD81 antibody	Abcam	MAB4863‐SP	WB, iEM
Europium nanoparticle	Invitrogen	603780	Immunoassay
Red assay buffer	Invitrogen	A14262J	Immunoassay
10 nm gold coated donkey‐anti‐mouse secondary antibody	Thermo‐Fisher Scientific	21787907	iEM
SDS laemmli sample buffer	Bio‐Rad	1610737	WB
4%–20% precast polyacrylamide gels, 12 wells, 20 µL	Bio‐Rad	#456‐1095	WB
PageRuler protein ladder	Thermo‐Fisher Scientific	27628663	WB
Nitrocellulose membrane	Sartorius Biotech	0823113272302233	WB
Donkey anti‐mouse secondary antibody, IRDye: 680 RD	LI‐COR	D30322‐25	WB
Donkey anti‐rabbit secondary antibody IRDye: 800 CW	LI‐COR	D41378‐80	WB

### 2.4 Transmission Electron Microscopy

TEM was performed to visualize EVs using a standardized negative staining protocol. To optimize image quality, two negative staining approaches were compared: (1) grid handling on Parafilm and (2) direct sample application using tweezers. The latter approach, combined with a reduced uranyl acetate concentration (1%) and shorter staining time (1 minute), yielded superior image contrast and lower background noise. This protocol was used for all subsequent imaging. Cu grid was plasma cleaned to make it hydrated using Targeo plasma cleaner (PIE Scientific). EV samples were first fixed in 2% paraformaldehyde (PFA) at room temperature for 2 hours. For grid preparation, 5 µL of the fixed sample was applied to carbon‐coated, glow‐discharged (water/air sources) 200‐mesh copper grids and incubated for 5 minutes. The grids were gently washed with filtered water and stained with 1% uranyl acetate for 1 minute. After staining, excess solution was removed, and the grids were air‐dried for 5 minutes. Imaging was performed using a transmission electron microscope (JEM‐1400, operating at 80 kV, with SIS Quemesa camera). Images were acquired using a CCD camera with live imaging set to a resolution of 1344×896 and snapshot quality mode at 2016×1344.

### 2.5 Immunostaining of EVs

Immunogold labelling was performed on EVs derived from both cell culture and patient serum to detect surface markers using TEM. Carbon‐coated 200‐mesh copper grids were treated with water/air plasma (glow discharge) to enhance hydrophilicity and facilitate EV adsorption. Fixed EV samples (5 µL, in 2% PFA) were placed on parafilm inside a humidity chamber composed of a moist paper towel and a foil‐wrapped dark box. The grids were positioned shiny side down onto the droplets and incubated for 20 minutes. After adsorption, grids were gently rinsed twice with droplets of 1xPBS and transferred to a blocking solution containing 1% bovine serum albumin (BSA) in PBS for 10 minutes. Following blocking, grids were washed 4 times with PBS and briefly air‐dried. Grids were then incubated with 5 µL of mouse anti‐human CD9 or CD81 primary antibody (1:100 dilution) for 45 minutes in separate labelling experiments. Control grids were incubated with blocking buffer only. After primary incubation, the grids were rinsed and then incubated with 5 µL of 10 nm gold‐conjugated donkey anti‐mouse secondary antibody (1:100 dilution) for 30 minutes. The grids were subsequently fixed with 1% glutaraldehyde, rinsed with PBS, and washed 7 times with Milli‐Q water. Finally, grids were stained with 1% neutral uranyl acetate for 1 minute and air‐dried for 5 minutes before imaging by TEM.

#### 2.5.1 Immunofluorescence Staining of DLBCL Cells

Immunofluorescent staining was performed to visualize the tetraspanins CD9, CD81 and CD63 presence in the cells. The non‐adherent DLBCL cells were plated on a‐IgM/IgG‐coated (10 µM/ml) coverslips or 12‐well cell imaging slides to facilitate cell adhesion via binding to their receptors (IgM for U‐2, Ri, O‐7 and IgG for S‐4). Cells were seeded in 0.5 % FBS/RPMI onto dishes and allowed to adhere for 45 minutes before fixing with 4% PFA (20’). The fixed cells were permeabilized with 0.1 % Triton X‐100 in 1%BSA/PBS (10’), blocked with 1%BSA/PBS (10’) and stained with conjugated antibodies 1:100 (in 1%BSA/PBS) a‐CD63‐488 and CD81‐560 (60’); separately with a‐CD9 1:100 (60’) followed by anti‐mouse Alexa Fluor 488 1:5000 (45’). The stained samples were washed and stained additionally with 1 µg/ml DAPI nuclear stain (1’) before mounting the samples with Fluoromount medium. The samples were imaged with 3i CSU‐W1 spinning‐disc confocal microscope using same exposure times for the same markers and cell samples. Maximum projections of the imaged cells were created with Fiji with brightness and contrast adjustments.

#### 2.5.2 Leprechaun Analysis of EV Samples

The tetraspanin expression analysis of cell line EVs and serum EVs isolated was performed independently at Unchained Labs according to their established protocols. The deep‐frozen supernatant or unprocessed serum samples were sent in dry ice to their laboratory. Briefly, the Lunis were coated with their pre‐optimized tetraspanin antibodies against CD9, CD81 and CD63 that capture the EVs expressing the respective antigens on the EV surface, from cell culture medium supernatants or directly from serum samples. The relative expression of the three tetraspanins were calculated size‐dependently in the samples using established protocols (data not shown in the manuscript).

### 2.6 Immunoassay

#### 2.6.1 Time Resolved Fluorescence Immunoassay (TRFIA)

A time‐resolved fluorescence‐based sandwich immunoassay was used to quantify the expression of tetraspanins CD63 and CD81 on EVs derived from both DLBCL cell lines (pooled EV samples or isolated fractions, when mentioned). Streptavidin‐coated 96‐well plates were washed and coated with biotinylated primary antibodies against CD63 or CD81 at a concentration of 100 ng per well in red assay buffer. Plates were incubated for 1 hour at room temperature with gentle agitation. Following incubation, the plates were washed to remove unbound antibodies. EV samples, diluted to a total protein of 3 µg per well in red assay buffer, were added and incubated for 1 hour at room temperature with slow shaking. Europium‐labelled monoclonal antibody‐conjugated nanoparticles were then added (2E7 nanoparticles per well) and incubated for 1.5 hours under gentle agitation. After four washes with wash buffer (Uniogen), time‐resolved fluorescence signals were measured using a plate reader with excitation and emission wavelengths of 340 nm and 615 nm, respectively. All measurements were performed in duplicate, and mean fluorescence values were calculated. Wash steps were performed using an automated plate washer to ensure consistency.

#### 2.6.2 Biotin Streptavidin Fluorescence Immunoassay (BSFIA)

To complement the immune assay, BSFIA method was employed for the detection of CD9 on EVs from both cell culture and serum samples. Flat‐bottom, high‐binding 96‐well plates were incubated with 2 µg of each EV sample overnight at 4°C. Due to the masking effect of other biomolecules onon serum‐derived EVs, the samples were first permeabilized using 0.1% Triton X‐100 (30’ on ice and vortexed) to increase the marker accessibility and antibody binding. The following day, plates were blocked with 300 µL of 1% BSA/TBST and incubated at room temperature for 2 hours. After washing (Washing buffer 42‐01, Uniogen), primary antibodies against CD9 (diluted 1:300 to 1:500 in 1% BSA/TBST) were added and incubated for 2 hours at room temperature. This was followed by sequential incubations with biotinylated secondary antibodies and europium nanoparticle‐coated streptavidin, each for 1 hour. After six washes with wash buffer, an enhancer solution was added and incubated for 45 minutes. Fluorescence intensity was measured using a time‐resolved fluorescence plate reader. This method improved signal detection, particularly in serum‐derived EVs where low marker abundance and surface masking by the protein corona may reduce detection sensitivity.

### 2.7 Western Blotting (WB)

WB was performed to analyse the presence of both surface and luminal EV‐associated proteins from cell line‐derived and patient serum‐derived EVs. When mentioned, EV samples were permeabilized with 0.1% Triton X‐100 (30’ on ice and vortexed) and supernatant EVs concentrated using SpeedVac (60’, Thermo Fisher). For WB, fractionated EV samples were loaded at 5 µg for serum‐derived EVs and 12 µg for cell line derived EVs, whereas pooled EV samples were loaded at 15 µg. All samples were mixed with 4× SDS Laemmli sample buffer (without β‐mercaptoethanol, BMe, unless specifically mentioned) and boiled at 95°C for 5 min prior to electrophoresis and membrane transfer. Cell lysates from DLBCL cell lines were used as controls; 5 µL of lysate per sample was loaded. Samples were run on 4%–20% precast polyacrylamide gels under non‐reducing conditions. Electrophoresis was conducted at 80 V during stacking and 120 V during separation for approximately 2 hours. Proteins were transferred to nitrocellulose membranes using the semi‐dry standard transfer protocol (Bio‐Rad, USA) at 100 V for 30 min at room temperature. Membranes were stained with Ponceau S (Sigma‐Aldrich) to verify equal protein loading and transfer efficiency, washed and blocked in 5% milk in TBST for 1 hour at room temperature. The following primary antibodies were used at previously optimized dilutions (in 1% BSA/PBS): CD9, CD81, CD44, and HSP70, CD19, CD20 and ApoA1. After overnight incubation at 4°C, membranes were washed with TBST and incubated with fluorescently labelled secondary antibodies for 1 hour at room temperature. Signal detection was performed using the Odyssey CLx imaging system (LI‐COR) under dual‐channel settings (700 and 800 nm).

### 2.8 Nanoparticle Tracking Analysis

NTA was used to measure the size distribution and concentration of EVs derived from DLBCL cell lines and patient serum. Complementary instruments were used to assess EV characteristics: ZetaView PMX‐120, NanoSight Pro and NS300.

#### 2.8.1 Analysis Using ZetaView PMX‐120

EVs were analysed using the ZetaView PMX‐120 system equipped with a 488 nm laser and a high‐sensitivity sCMOS camera at EV core facility (University of Helsinki, Finland). Prior to analysis, concentrated EV samples were diluted in filtered 1× PBS (0.1 µm filtered) to reach an optimal particle concentration. The instrument was calibrated using polystyrene beads, and measurements were taken at 11 positions, tracking 50‐200 particles per frame over three measurement cycles under a controlled temperature of 22°C, with data collected at a laser power of 42 mW, camera gain set to 33.6, camera sensitivity to 85, and shutter speed to 100. Three 30‐second video recordings were acquired per sample and analysed using ZetaView software (version 8.05.16 SP3). The system supports particle detection in the range of 70–2000 nm and concentrations between 1×10^5^ and 1×10^9^ particles/ml. To facilitate downstream interpretation, raw data were processed and visualized using OriginLab (2016), where signal smoothing was applied using the Savitzky–Golay filter with a window size of 15 to improve peak definition in the distribution graphs.

#### 2.8.2 Analysis Using NanoSight Pro and NS300

EVs isolated from patient serum were additionally characterized using the NanoSight Pro during a demo and NS300 (Malvern Panalytical; data not shown) systems, high‐resolution NTA instruments equipped with a 488 nm laser and an ultra‐sensitive sCMOS camera that is capable of detecting particles in the approximate size range of 30–1000 nm. EV samples were diluted in 0.1 µm‐filtered Milli‐Q water to achieve a target concentration of 1×10^8^‐1×10^9^ particles/ml. Measurements (Nanosight Pro) were performed at room temperature using standard acquisition settings, with one replicate capture including five runs per sample (60 seconds each). Additionally, two of the four patient samples were analyzed with three replicate captures (NS300) with comparable or non‐comparable size distributions using both devices. Data were analysed using NS XPLORER v1.1.0.6 or NTA 3.4 Build 3.4.4 softwares.

### 2.9 MS Analysis

MS analysis was conducted at the Turku Proteomics Facility to characterize the protein content of SEC‐purified EVs derived from both cell culture and patient serum. A data‐independent acquisition (DIA) LC‐MS/MS approach was used for quantitative proteomics. EV samples were lysed in a buffer containing 8 M urea, 0.5% NP‐40, 150 mM NaCl, and protease inhibitors, followed by incubation on ice for 30 minutes. The lysates were sonicated using a 30s on/30s off cycle for 5 minutes on Bioruptor+ (Diagenode). Proteins were then precipitated by adding four to five volumes of cold acetone and incubated overnight at ‐20°C. Proteins were pelleted by centrifugation at 16,000x g for 15 minutes at 4°C, and dissolved in 8 M urea, 50 mM ammonium bicarbonate buffer. Protein disulfide bonds were reduced with 10 mM dithiothreitol (DTT) at 37°C for 1 hour and alkylated with 40 mM iodoacetamide (IAA) in the dark for 1 hour. The reaction was quenched by adding an additional 40 mM DTT. The urea concentration was diluted to <1.5M, and samples were digested overnight with sequencing‐grade trypsin at a 1:30 enzyme‐to‐protein ratio at 37°C. Digestion was stopped by acidification with 1% trifluoroacetic acid (TFA), and peptides were desalted using C18 solid‐phase extraction and dried by vacuum centrifugation.

Dried peptides were reconstituted in 0.1% formic acid, and 600 ng of peptides per sample were loaded onto Evotip Pure tips for injection into an Evosep One LC system. Peptides were separated using a 150µm x 15 cm C18 analytical column with 1.5µm beads (PepSep/Bruker) with the 30 samples per day method. The mobile phases consisted of 0.1% formic acid in water (solvent A) and 0.1% formic acid in 99.9% acetonitrile (solvent B). Eluted peptides were analysed on a timsTOF fleX MALDI‐2 mass spectrometer equipped with a CaptiveSpray nano‐electrospray ionization source. DIA acquisition was performed using dia‐PASEF mode, covering an m/z range of 350–1100. Twenty‐five dia‐PASEF scans were acquired per sample, with ion mobility (IM) windows set at 1.3 and 0.6 V·cm^−^^2^, and an accumulation/ramp time of 100 ms. Raw data were processed using Spectronaut software with a DirectDIA workflow. Protein identification was performed using a human UniProt database, and label‐free quantification was conducted using MaxLFQ. Search parameters included trypsin/P as the enzyme, allowance of up to two missed cleavages, carbamidomethylation (C) as a fixed modification, and acetylation (protein N‐term) and oxidation (M) as variable modifications. Both precursor and protein false discovery rates (FDR) were controlled at 1%.

For the automated magnetic enrichment based on charge of lipid particles from the samples, a protocol essentially following Wu et al. (2025), was followed. Briefly, 20 ul of serum samples were mixed with equal volume of binding buffer to a final volume of 50 µL. Then, 2 µL of MagReSyn SAX beads (ReSyn Biosciences) were added and mixed by pipetting. Reduction, alkylation, and wash steps were performed using an Opentrons pipetting robot (Opentrons, New York, United States) following the MagNet enrichment protocol. In the final step, sequencing‐grade modified trypsin (Promega) was added, and the sample plate was incubated overnight at +37°C. On the following day, half of each sample was loaded onto Evotip Pure tips (Evosep) and analysed with the same LC‐MS method as the SEC‐purified EVs.

### 2.10 Statistical and Image Analysis

Statistical analyses were performed to evaluate the significance of experimental results obtained from EV characterization assays. Comparative analyses were conducted using paired t‐tests, one way ANOVA, and two‐way ANOVA, depending on the experimental design. For MS data analysis, one‐way ANOVA and data normalization were performed. Data were normalized by setting the lowest intensity value to 0 and the highest value to 100, enabling comparative analysis comprising heatmap and graph preparation. *p*‐values were interpreted as follows: *p* ≤ 0.05 (*), *p* ≤ 0.01 (**), *p* ≤ 0.001 (***) and *p* ≤ 0.0001 (****). All statistical calculations, data normalization, and graph generation were carried out using GraphPad Prism (version 10.2), Microsoft Excel, and OriginLab (2016). For image quantification, ImageJ software was used to measure particle diameter. Scientific illustrations were generated using BioRender and figure layout and graphical annotations were finalized using CorelDraw (version 10). Unless otherwise specified in figure legends, all error bars represent mean ± standard deviation.

### 2.11 Antibodies, Reagents and Materials (Table 3)

## 3 Results

### 3.1 EV Characterization

**FIGURE 1:**
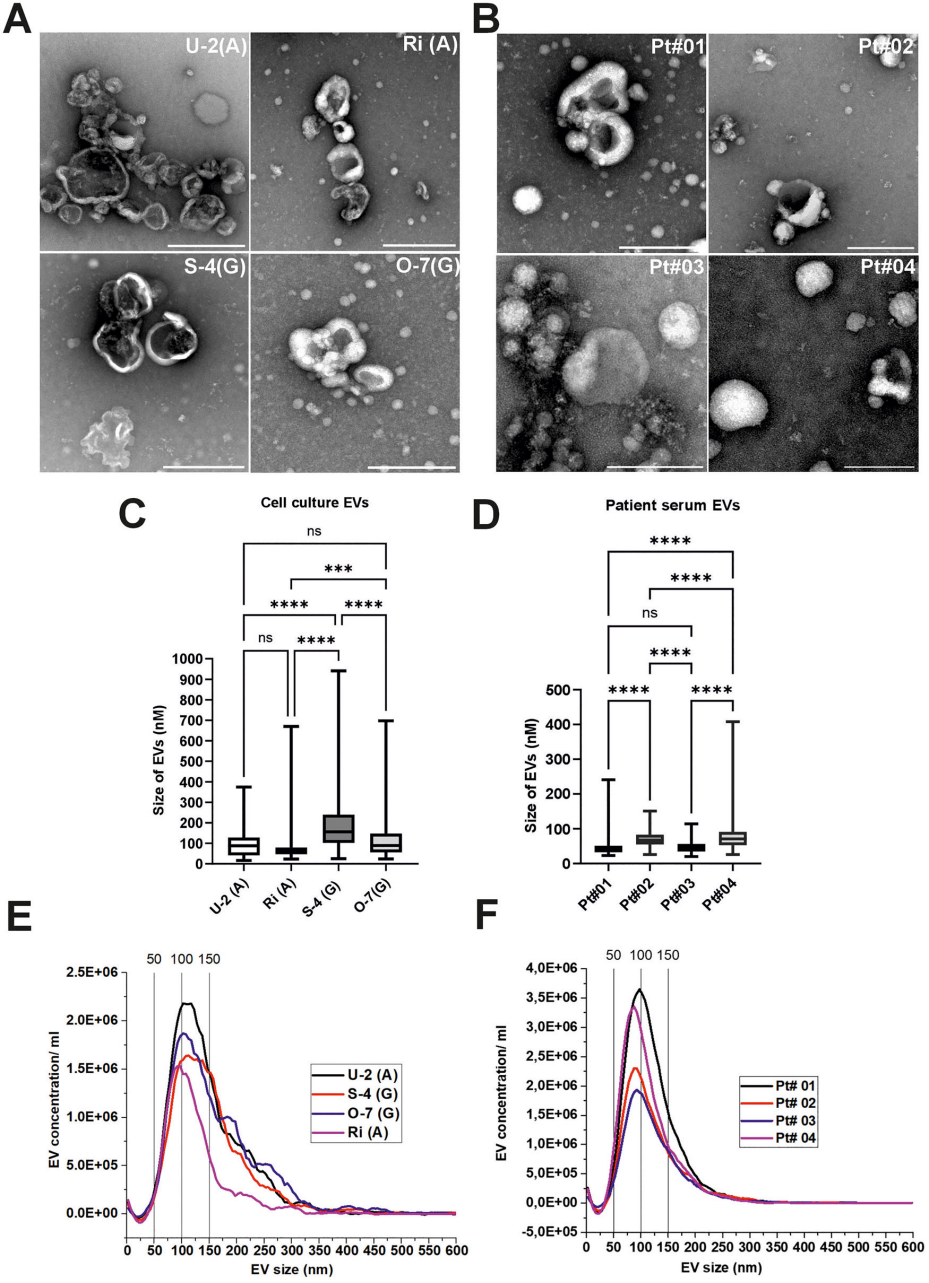
Morphological and size‐based characterization of EVs from DLBCL cell lines and lymphoma patients’ serum. (A) TEM images of negatively stained EVs isolated from ABC‐subtype (U‐2, Ri) and GCB‐subtype (S‐4, O‐7) DLBCL cell lines, showing typical cup‐shaped and round morphologies. (B) TEM images of EVs isolated from serum samples of four BCL patients (Pt#1–Pt#4), highlighting inter‐patient variability in EV morphology and abundance. (C) Manual size distribution of cell line‐derived EVs shows that ABC‐type EVs (U‐2, Ri) are smaller and more abundant than GCB‐type EVs (S‐4, O‐7). (D) Size measurements of patient‐derived EVs demonstrate significant heterogeneity across individuals. (E) NTA curves of EVs from DLBCL cell lines reveal subtype‐dependent size distributions, with ABC lines producing smaller particles. (F) NTA profiles of patient‐derived EVs further confirm sample‐specific EV size and concentration difference. Scale bar for TEM images: 100 nm. Statistical significance: ns (not significant), *p ≤ 0.05, **p ≤ 0.01, ***p ≤ 0.001, ****p ≤ 0.0001.

TEM was employed to assess the quality of EV preparations from both DLBCL cell lines and patient serum. The EVs from cell culture displayed typical cup‐shaped forms. In contrast, serum‐derived EVs predominantly exhibited a round shape with more uniform borders. Notably, differences in EV morphology and abundance were observed across DLBCL subtypes. ABC subtype (U‐2, Ri) displayed smaller, more abundant EVs compared to the GCB subtype (S‐4, O‐7), which generated larger but less numerous vesicles (Figure 1A). Serum‐derived EVs (Figure 1B) were more variably between patient samples. It is important to note that we used pooled samples from SEC columns in most of the experiments and the presence of other particles such as lipoproteins within the samples is likely. To enrich the tetraspanin‐positive EVs from SEC columns and to exclude the fractions containing the most soluble proteins and lipoproteins from downstream analyses, we performed a series of experiments to the isolated fractions (Supplementary Figure 1). Based on the immunoassays, TEM and Western Blot analysis on fractions F1‐F4 we concluded that the first three fractions F1‐F3 were the most enriched with tetraspanins CD9, CD81 and CD63 in cell lines supernatants and in serum‐isolated EVs (Supplementary Figure 1A, B, D‐G) while the fraction F4 was observed with high‐abundance soluble protein in TEM (Supplementary Figure 1C: from U‐2 cells) and in total protein stain Ponceau S (Supplementary Figure 1D‐G). Also, the lipoprotein marker Apolipoprotein AI was most abundant in the F4 of the serum‐derived samples (Supplementary Figure 1F&G), though it must be noted that the presence of it was detected moderately in all fractions and samples and thereby we acknowledge the presence of other particles besides EVs equally in all samples. To be able to analyse the majority of the tetraspanin‐rich EVs with a reasonable sample yield, while still excluding the fraction with the most abundant soluble protein and lipoprotein contaminant, we decided to collect the fractions F1‐F3 in the further downstream analytics.

ABC‐derived EVs were smaller, with U‐2 EVs ranging from 30–170 nm and Ri EVs from 40–80 nm (Figure 1C). No statistically significant difference was detected between these two lines. GCB‐derived EVs demonstrated larger size distributions: S‐4 EVs spanned 100–250 nm, significantly larger than EVs from U‐2, Ri, and O‐7 (*p* ≤ 0.01). O‐7 EVs were similar in size to U‐2 but differed significantly from Ri and S‐4 EVs (Figure 1C). Serum‐derived EVs exhibited also considerable patient‐to‐patient heterogeneity in size (Figure 1D). For example, EV sizes in patients 1, 2, 3, and 4 ranged from 20–100 nm, with predominant distributions of 20–50 nm, 50–85 nm, 50–65 nm, and 60–100 nm, respectively. This heterogeneity highlights individual variation in circulating EV profiles among lymphoma patients, (Figures 1D).

NTA analysis (ZetaView PMX‐120) of cell line EVs showed that a median diameter of U‐2, Ri, S‐4 and O‐7 EVs was 128.5, 106.5, 143.9 and 125.6 nm, respectively; however, the total major range of particles was between ≈70–340 nm in the samples, and GCB‐derived EVs displayed broader and higher size profiles. In terms of concentration, EVs from ABC cell lines Ri and U‐2 had average particle counts of 8.7 × 10^1^ ^0^ and 7.1 × 10^1^ ^0^ particles/ml, respectively, compared to 2.8 × 10^1^ ^0^ (S‐4) and 2.0 × 10^1^ ^0^ (O‐7) particles/ml from GCB cell lines.

Serum‐derived EVs were found to be significantly smaller (TEM imaging) and their sizes were assessed with ZetaView PMX‐120 (Figure 1F). Median diameter of serum EVs of patients 1, 2, 3, and 4 were 107, 105.6, 107.3 and 97.1 nm. Total particle concentrations for serum EVs 1, 2, 3, and 4 were 6.6 × 10^1^ ^0^, 8.3 × 10^11^, 3.6 × 10^1^ ^0^ and 2.8 × 10^11^ particles/ml, respectively. In conclusion, the NTA analyses showed the same trends in general EV sizes as was detected in manual measurement based on TEM images (Figures 1C&D and 1E&F), although the average sizes of EVs between these techniques varied a lot. These findings highlight the complexity of evaluating the sizes in EV preparations.

### 3.2 Detection of EV Surface Markers by Immunogold TEM

**FIGURE 2:**
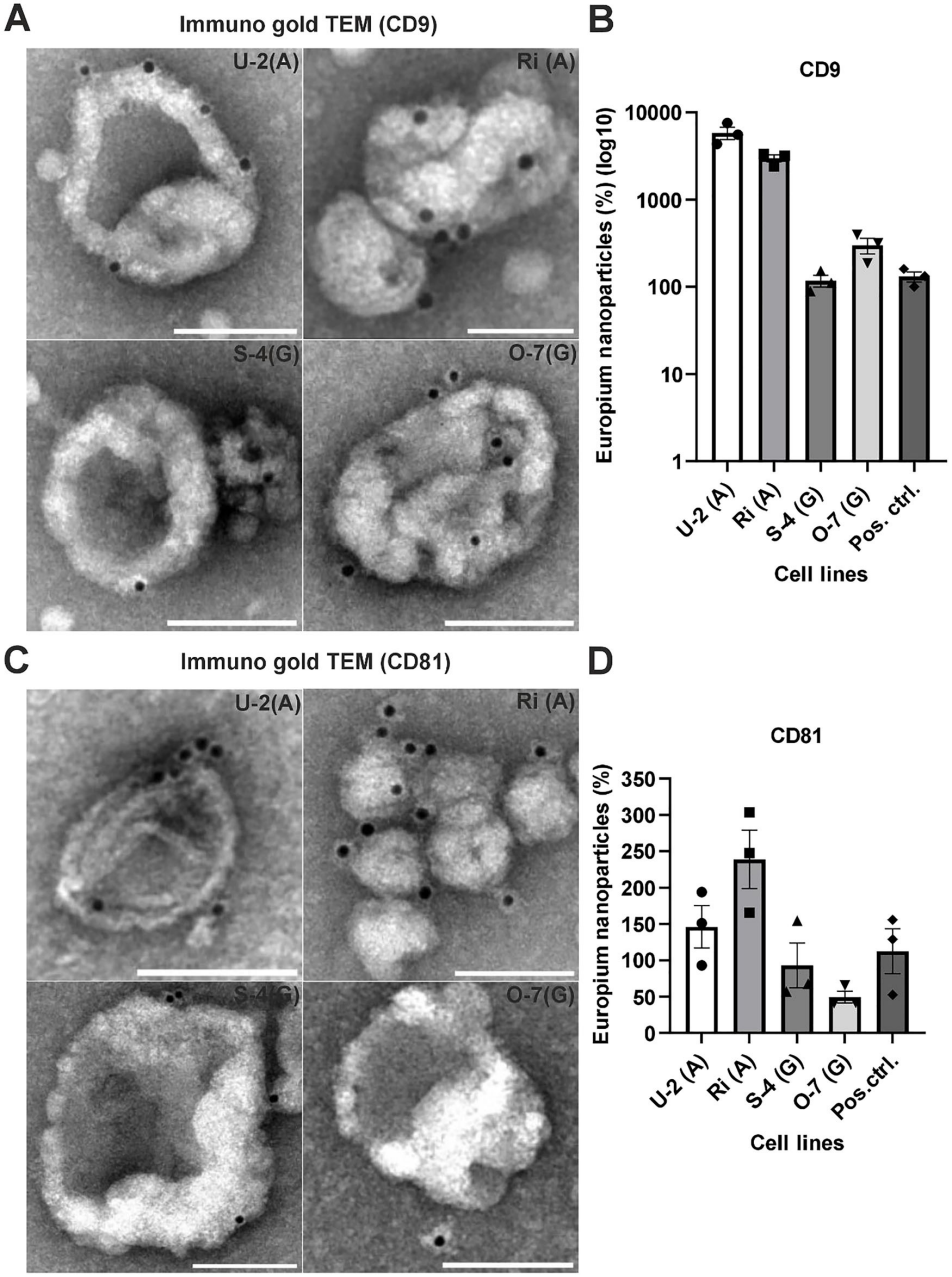
EV marker profiling by immunogold TEM and europium nanoparticle‐based immunoassays. (A) Immunogold TEM images of EVs isolated from ABC‐type (U‐2, Ri) and GCB‐type (S‐4, O‐7) DLBCL cell lines, labeled with antibodies against CD9. (B) Quantification of CD9 expression on cell line‐derived EVs using europium nanoparticle‐based assays. The SEC‐isolated pooled fractions F1‐F3 were analysed for relative tetraspanin expression in different cell types. Graphs present the average of 3 individual experiment values; error bars represent SEM values. (C) Immunogold TEM images of EVs isolated from ABC‐type (U‐2, Ri) and GCB‐type (S‐4, O‐7) DLBCL cell lines, labeled with antibodies against CD81. (D) CD81 expression quantification on cell line‐derived EVs using europium nanoparticle‐based assays. CD9 was measured via biotin streptavidin fluorescence immunoassay (BSFIA), while CD81 were assessed by time‐resolved immunofluorometric assay (TRFIA). Y‐axis in panels B is shown on a log10 scale. All data represent the mean from three independent experiments. Scale bars in TEM images = 100 nm.

Immunogold TEM (iEM) was used to localize key EV surface markers (CD9 and CD81) on vesicles derived from DLBCL cell lines and patient serum. EVs from ABC subtype cell lines (U‐2 and Ri) exhibited clear gold particle labelling for both CD9 and CD81, confirming the presence of these markers on the EV surface (Figure 2A&C). Among GCB subtypes, S‐4 EVs showed visible but less intense labelling, while O‐7 EVs displayed the weakest staining, suggesting lower surface marker expression (Figure 2A&C). Control grids (not shown here) incubated with blocking buffer and secondary antibody showed no background signal.

In contrast, serum‐derived EVs demonstrated non‐functional labelling for CD9, CD81 or any other tested markers using the staining protocol that was used with cell line EVs (data not shown). We attempted permeabilization of patient‐derived EVs 0.1% Triton X‐100, however, this process caused vesicles to detach from the EM grids, preventing successful imaging. These findings highlight both the technical limitations of using the same experimentation as iEM for serum‐derived EVs or cell line‐derived vesicles.

### 3.3 Tetraspanin Expression in DLBCL Subtypes: Immunoassay‐Based Analysis

Quantitative analysis of EV markers was conducted using TRFIA and biotin streptavidin fluorescence immunoassay (BSFIA) for CD9, CD81 and CD63. All measurements were performed on EVs isolated from U‐2, Ri, S‐4, and O‐7 cell lines and for serum‐derived EVs (for CD9), and normalized to protein concentration. Prostate cancer cell line DU145 EVs were used as a positive control for tetraspanin expression (Figure 2B&D; Supplementary Figure 2A&B; Islam et al., 2019).

In BSFIA assay, CD9 was detected in all DLBCL‐derived EVs, though with varying expression level (Figure 2B). CD81 levels were notably higher than CD63 across all samples, with ABC‐type EVs (especially Ri) displaying the strongest expression (Figure 2D). Within the GCB‐derived EVs, S‐4 EVs expressed higher levels of CD81 than O‐7 EVs. CD63 levels were consistently lower in all samples as compared to CD9 and CD81 (Figure 2B&D; Supplementary Figure 2A), although Ri EVs still maintained relatively high expression Supplementary Figure 2A). Collectively, the immunoassay data highlight a pattern of elevated CD81 and CD9 expression in ABC subtype EVs in comparison to GCB EVs. Furthermore, these findings were reinforced with outsourced Leprechaun analysis (Unchained Labs, data not shown) in which a direct chip‐bound antibody capture of CD81, CD63 and CD9 ‐positive EVs is employed. According to the analysis, all the three tetraspanins were expressed at least 10‐fold more in ABC cell EVs (Ri and U‐2) than in GCB cell EVs (S‐4 or O‐7), aligning with our other data expressing the whole vesicle population. In serum‐derived EVs analysed with the same technique, all the three tetraspanins were modestly though variably expressed in all 4 samples. Interestingly, when observed with immunofluorescence staining, the tetraspanins CD63, CD9 and CD81 were all equally expressed within all the cell lines with no obvious difference in marker expression between the ABC and GCB cell lines (Supplementary Figure 2C). CD9 expression was also measured in serum‐derived EVs (0.1 % Triton‐X 100 permeabilized) using BSFIA; CD9 was found present in all four samples, with differential and modest expression (Supplementary Figure 2B). These results, together with the iEM findings, suggest that immunoassays may be more sensitive for detecting surface markers in clinical serum‐derived EVs than microscopy‐based techniques.

### 3.4 Immunogold EM Reveals Size‐Dependent Marker Expression on Small and Large DLBCL EVs

**FIGURE 3:**
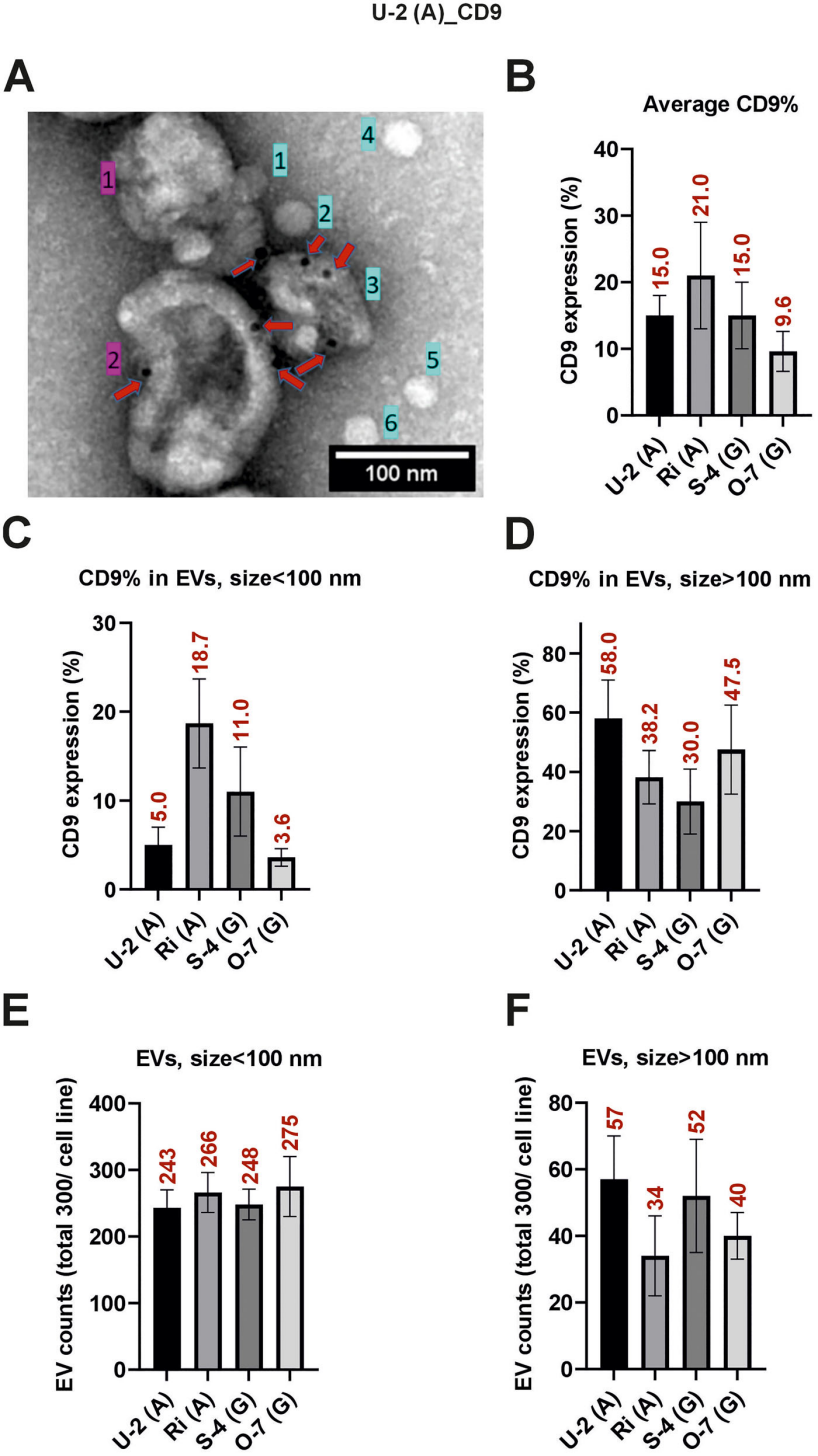
Size‐dependent analysis of CD9 expression in EVs from DLBCL cell lines using immunogold TEM. (A) Representative TEM image of CD9‐labeled EVs from U‐2 (ABC subtype) showing surface localization of gold particles (red arrows). EVs larger than 100 nm are marked in magenta; vesicles and particles smaller than 100 nm are marked in cyan. (B) Average percentage of CD9‐positive EVs across all size ranges in four DLBCL cell lines (U‐2, Ri, S‐4, O‐7). (C, D) Size‐stratified analysis showing the proportion of CD9‐positive EVs in the <100 nm (C) and >100 nm (D) EV subpopulations. (E, F) Total number of EVs measured per group for the corresponding size categories: <100 nm (E) and >100 nm (F). ABC‐type EVs (U‐2, Ri) showed stronger CD9 positivity, particularly in the >100 nm population. Data represent the mean from three independent experiments (total n = 300 n = 100 per experiment). Scale bar = 100 nm.

To further characterize the EVs derived from cell lines, we performed immunogold EM (iEM) to visualize and quantify the surface expression of CD9 and CD81. Representative TEM images of U‐2 EVs are shown for both CD9 and CD81 immunostaining (Figure 3A and Supplementary Figure 3A), while the quantification includes EVs from all four DLBCL cell lines U‐2 and Ri (ABC subtype), and S‐4 and O‐7 (GCB subtype). The EVs were categorized into two size‐based populations: small (<100 nm) and large (>100 nm), to evaluate marker distribution across EV sizes. Quantitative analysis revealed that the overall proportion of CD9‐positive EVs varied across the cell lines, with the highest expression observed in Ri EVs (21.0%), followed by U‐2 and S‐4 (both 15.0%), and the lowest in O‐7 (9.6%) (Figure 3B). When stratified by EV size, CD9 was more frequently detected on larger EVs (>100 nm) than on smaller ones (<100 nm). For instance, in U‐2‐derived EVs, CD9 was present in 58.0% of large EVs compared to only 5.0% of small EVs (Figures 3C‐F). This trend was consistent across all cell lines, suggesting a size‐dependent enrichment of CD9 into the larger EVs in DLBCL. Though, it must be noted that the small‐sized population contains other particles than merely tetraspanin‐positive EVs and this may deviate the result in that sized population.

Similarly, CD81 expression was highest in ABC subtype EVs, with U‐2 EVs showing 61.5% positivity, followed by Ri (40.7%). GCB‐derived EVs showed lower CD81 positivity, with S‐4 at 18.3% and O‐7 at 16.0% (Supplementary Figure 3A&B). CD81, like CD9, was predominantly enriched in the larger EV population (>100 nm), with U‐2 EVs showing 70.2% positivity in this size group (Supplementary Figure 3C). CD81 detection in smaller EVs was noticeably lower, particularly in GCB‐derived EVs (9.6%–12.3 %; Supplementary Figure 3D‐F). Interestingly, EVs derived from the ABC subtype exhibited relatively high expression of both CD9 and CD81 overall. However, the distribution of these markers varied slightly between them. For instance, S‐4 EVs showed elevated levels of CD63 (based on earlier results) and CD9, but comparatively low CD81 expression. This suggests potential subtype‐specific heterogeneity in vesicle marker composition. EV counts across size categories showed that small EVs (<100 nm) were more abundant than large EVs (>100 nm) in all cell lines (Figures 3E&F; Supplementary Figure 3E&F), but the larger EVs consistently carried a higher percentage of detectable markers.

These data demonstrate a cell subtype (or source)‐ and size‐dependent pattern of tetraspanin expression in DLBCL‐derived EVs. The strong enrichment of CD81 and CD9 in ABC cell‐derived large EVs suggests that vesicle surface composition may reflect underlying molecular differences between DLBCL subtypes.

### 3.5 Proteomic Profiling of DLBCL Cell Line and Serum‐Derived EVs

**FIGURE 4:**
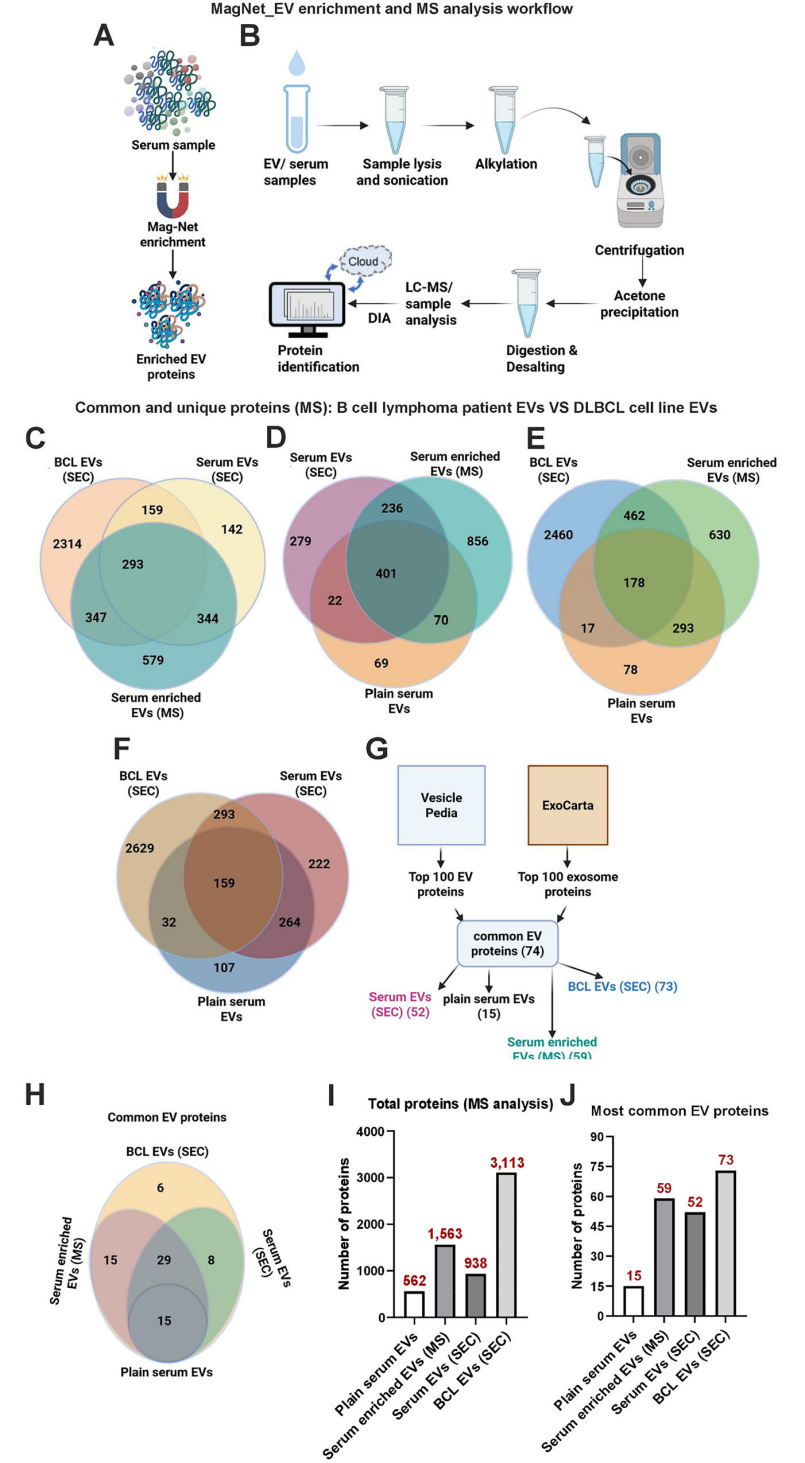
Comparative proteomic profiling of EVs from DLBCL cell lines and patient serum. (A) EV enrichment using Mag‐Net from patient serum sample. (B) Schematic work flow of MS analysis of SEC isolated EVs and plain serum samples (C‐F) Venn diagrams illustrate the overlap of proteins identified by DIA‐MS in four sample types: SEC‐isolated EVs from DLBCL cell lines, SEC‐isolated EVs from patient serum, serum‐enriched EVs (MS), and plain serum. (C) Comparison of EV‐associated proteins between SEC‐isolated BCL EVs, SEC‐isolated serum EVs, and serum‐enriched EVs (MS). (D‐E) Inclusion of plain serum highlights the presence of background proteins compared to EV‐specific signals in SEC or MS‐enriched fractions. (F) Full comparison across all four sample types reveals 159 proteins shared between SEC‐isolated BCL EVs and serum EVs. (G) Mapping of top 100 EV markers from Vesiclepedia and ExoCarta databases across sample types. SEC‐isolated BCL EVs retained the greatest number of canonical markers. (H) Venn diagram of the 74 most commonly reported EV proteins, showing distribution across sample types. (I) Total number of proteins identified per sample by MS (J) Number of top EV‐associated proteins detected per sample. *Graphical illustrations created in BioRender. Hämälistö, S. (2026)* https://BioRender.com/e3vg0s7;BioRender.com/g0j4ter;BioRender.com/k2zb36i;BioRender.com/kbucmuf.

We performed MS‐based proteomics characterization of the following samples: (1) SEC‐isolated BCL EVs, (2) SEC‐isolated serum EVs, (3) MagNet‐enriched serum EVs (Figure 4A), and (4) plain serum (see Figure 4B). Across all sample types, we identified 5269 proteins. The lists of identified proteins are found as Supplementary Files 1–4 (1: SEC‐isolated DLBCL EVs, 2: SEC‐isolated serum EVs, 3: MagNet‐enriched serum EVs, 4: plain serum). MS confirmed a broad repertoire of previously reported EV‐associated proteins (e.g., CD9, Alix, Hsps) in both cell line and serum EVs, with cell line EVs showing more protein identifications than serum‐derived EVs. MagNet enrichment of serum samples allow direct EV analysis by MS without separate EV isolation (Wu et al. (2025); Figure 4A&B). SEC‐isolated BCL EVs yielded the highest number (3113), followed by serum‐enriched EVs (1563), SEC‐isolated serum EVs (938), and plain serum (562) (Figure 4I). This highlights the varying efficiencies between sample matrices and superior enrichment and depth of proteome coverage achieved by SEC. Venn diagram comparisons revealed the extent of protein overlap and uniqueness among the groups. A total of 293 proteins were common to SEC‐isolated BCL EVs, SEC‐isolated serum EVs and MagNet‐enriched serum EVs (Figure 4C). An additional 401 proteins were shared across SEC‐isolated serum EVs, serum‐enriched EVs, and plain serum, while 236 proteins were unique to SEC‐isolated serum EVs alone (Figure 4D). Compared to plain serum, 178 proteins were consistently detected across all EV‐containing fractions (Figure 4E&F), suggesting the presence of a core EV proteome irrespective of the sample source.

To assess EV specificity, we compared our datasets to the top 100 EV‐associated proteins curated from Vesiclepedia and ExoCarta (Figure 4G). Seventy‐four of these canonical EV proteins were common in the two databases. Compared to this, 74 EV proteins with the greatest number detected in SEC‐isolated BCL EVs (73), followed by serum‐enriched EVs (59), SEC‐isolated serum EVs (52), and plain serum (15) (Figure 4J). This confirms that EV marker enrichment is most efficient in SEC‐isolated vesicles from cell culture supernatants. Canonical EV markers including CD9, CD81, HSP70, HSP90B1, Alix, and Ezrin were readily detected in EV‐rich samples but were largely absent in plain serum, confirming effective EV enrichment and marker specificity.

Further analysis of the 74 EV markers showed distinct and overlapping profiles across the four sample groups (Figure 4H). While 15 EV markers were shared in all preparations, 44 were common among serum‐enriched EVs, SEC‐isolated serum EVs, and SEC‐isolated BCL EVs. Only 6 markers were uniquely identified in BCL EVs, suggesting both method‐specific and source‐specific differences in EV protein content.

Heatmap analysis (Supplementary Figure 4) illustrates relative abundance differences of key EV‐associated proteins across serum and cell line samples. Proteins such as HSP90 isoforms, annexins, and syntenin‐1 showed high expression in cell line‐derived EVs, while serum EVs demonstrated more variability and lower intensities, likely reflecting biological heterogeneity and reduced EV yield from serum. Interestingly, the expression of CD44 and Gal3BP (galectin‐3 binding protein), both linked to B cell lymphoma or malignant progression and found in serum, was highly abundant across all the studied lymphoma cell lines and lymphoma patient‐derived EVs (Figure 5A; Giansanti et al., 2019; Tzankov et al., 2003). Quantitative analysis of tetraspanins and heat shock proteins on the other hand revealed that CD9 was more highly expressed in serum‐enriched EVs, whereas CD81 and HSP90B1 were consistently detected in both serum‐ and cell‐derived EVs (Figure 5A‐G). These results suggest differential expression or accessibility of common EV markers depending on sample origin and enrichment method.

FIGURE 5Proteomic quantification and WB validation of EV‐associated markers in DLBCL‐derived EVs. (A) Heatmap of normalized protein abundance for selected EV markers identified via DIA‐MS in SEC‐isolated EVs from four DLBCL cell lines (U‐2, Ri, S‐4, O‐7) and four BCL patient serum samples (Pt#01–Pt#04). (B‐C) MS‐based quantification of CD9 (B) and HSP90B1 (C) in SEC‐isolated serum EVs from four patients. (D‐E) MS quantification of CD9 (D) and HSP90B1 (E) in EV‐enriched serum pellets (MS preps) from four additional patients. (F‐G) MS‐based comparison of CD9 (F) and HSP90B1 (G) expression in SEC‐isolated EVs from DLBCL cell lines. (H‐J) WB validation of selected surface marker CD9, B cell specific markers (CD19, CD20), and luminal marker HSP70in SEC‐isolated EVs under non‐permeabilized, permeabilized, and on‐denaturing conditions. In Figure 5J, the two different SEC‐isolated samples were treated or not with beta‐mercaptoethanol reducing agent and blotted against markers CD9 and CD19. Cell lysates and ladder were included as controls.

**Figure:**
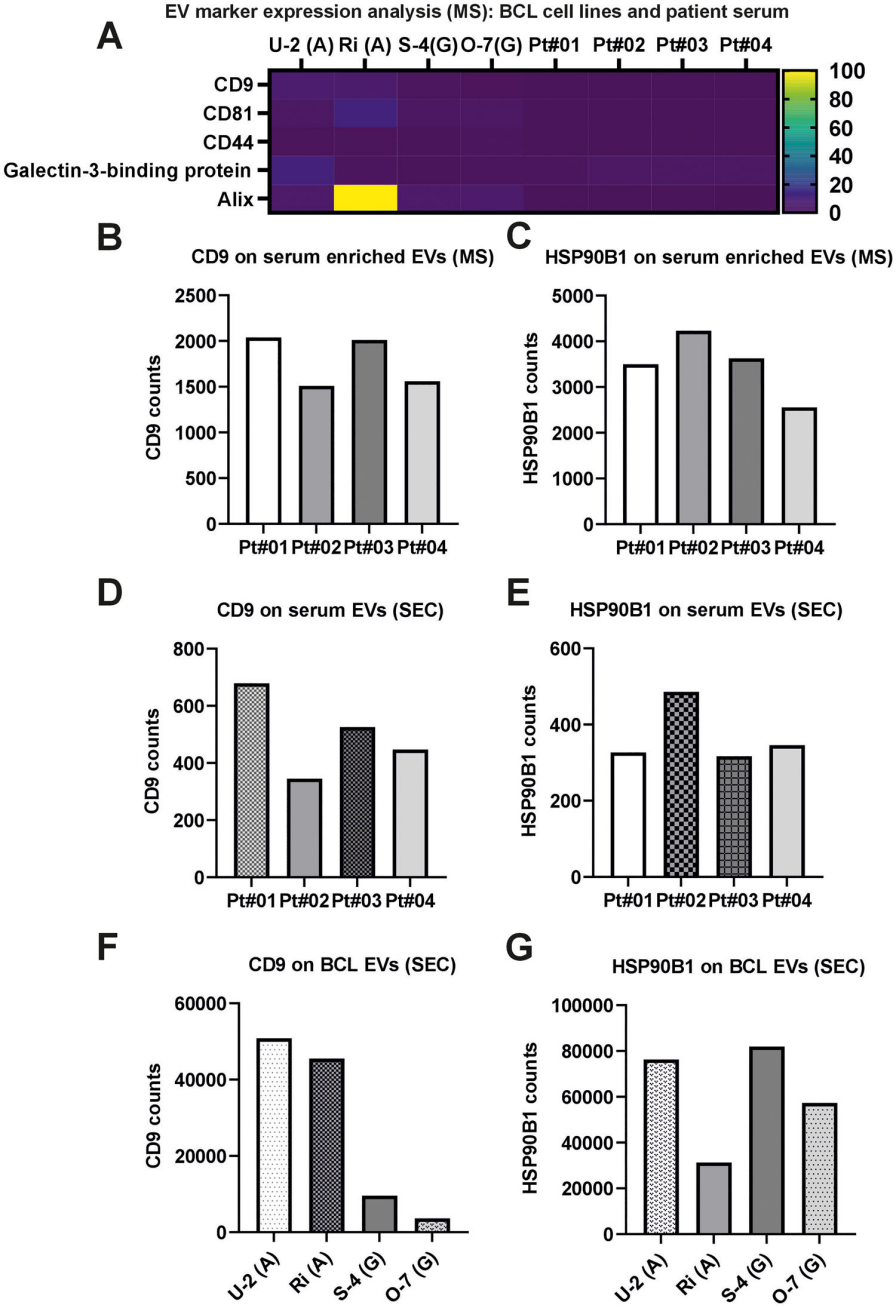

**Figure:**
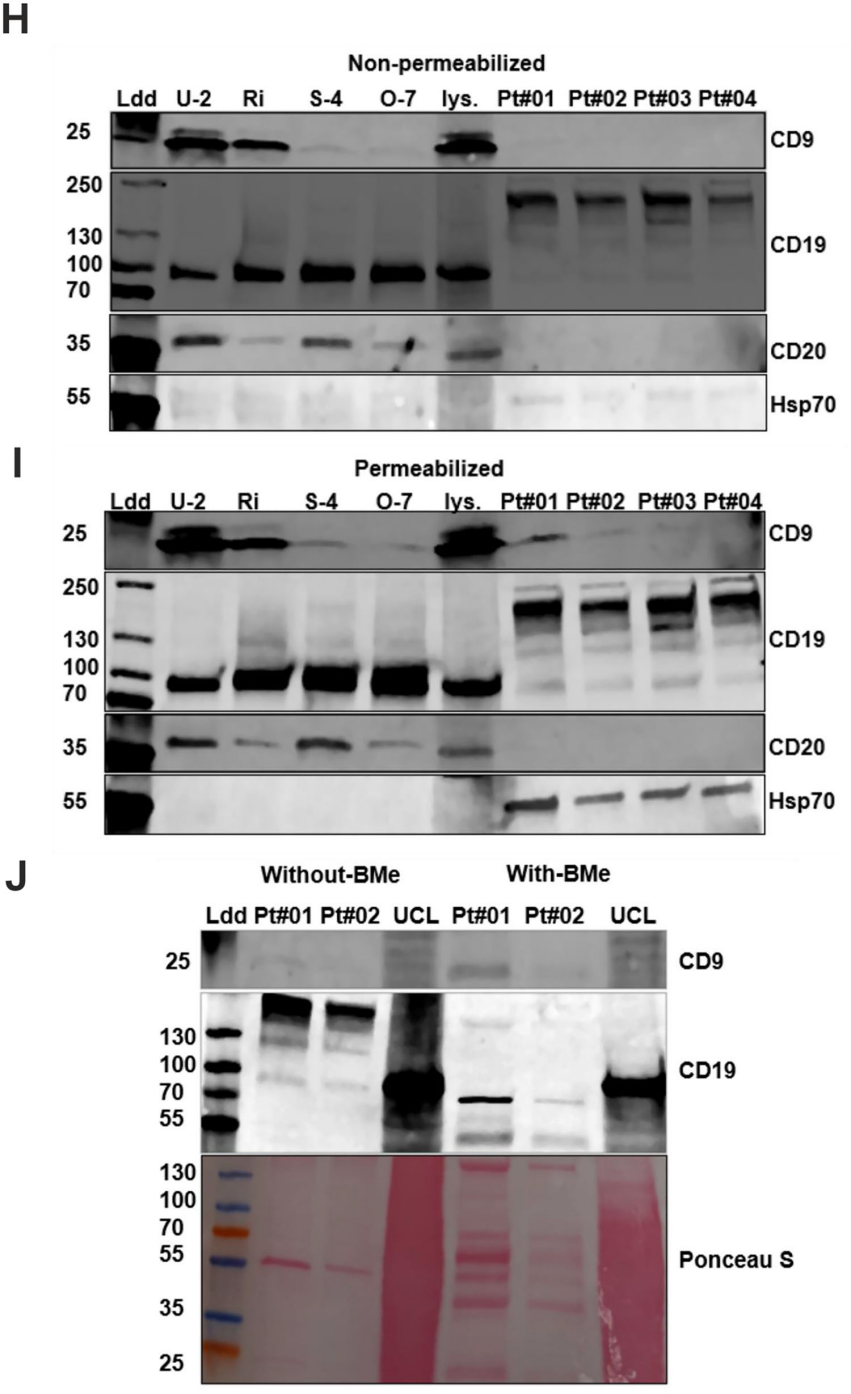

Altogether, our data demonstrate that DIA‐MS enables robust EV proteomic profiling and that SEC provides efficient EV enrichment, particularly from cultured lymphoma cells. Comparative analysis revealed distinct EV proteomic signatures within patient and cell line derived samples, offering valuable insight for biomarker discovery and mechanistic studies in lymphoma EV research.

### 3.6 Analysis of Essential Markers From BCL Cell Lines and Patient Serum Samples

To assess the expression of membranous and internal EV markers, WB analysis was performed on SEC‐isolated EVs from ABC type (U‐2, Ri) and GCB type (S‐4, O‐7) cell lines and serum EVs of four lymphoma patients (Pt#01‐Pt#04); Figure 5H‐J. The cell line‐derived EVs generally did not require permeabilization to improve canonical marker expression, and tetraspanins CD9 and CD81 were equally detected in non‐permeabilized and Triton‐X‐100 permeabilized samples. Interestingly, as shown for immunoassays and iEM in Figure 2, the ABC type U‐2 and Ri EVs showed relatively higher expression of tetraspanins compared to the GCB types S‐4 and O‐7. In serum‐derived EVs on the other hand, the tetraspanins were undetectable without permeabilization, which improved the CD9 signal intensity to a detectable level (Figure 5H&I): the CD81 signal was barely detectable in serum EV samples even after permeabilization step (Supplementary Figure 5A&B). Similarly, the Hsp70 and CD44 signal were nearly undetectable in the blots without the sample permeabilization step (Figure 5H&I; Supplementary Figure 5B).

Importantly, B cell‐specific markers CD19 and CD20 were clearly visible in cell line EVs and upon permeabilization the CD19 expression became more prominent also in serum‐derived EVs (Figure 5H&I). Additionally, in cell line EVs, a prominent CD19 expression was detected at a reported size of ∼95 kDa (Figure 5H&I). Interestingly, patient serum EVs expressed primarily a prominent ∼200 kDa size of CD19, reflecting a possible dimer formation in serum‐derived EVs. This hypothesis was strengthened by Western Blot experiments where the serum‐derived samples were treated or not with a reducing agent beta‐mercaptoethanol (β‐ME) to rupture the sulphur bridges between proteins: this treatment altered the CD19 signal to the reported size of ∼95 kDa (Figure 5J). This poses an interesting question of the form of CD19 being secreted into or expressed in the serum EVs, as compared to EVs derived from cell lines. Expression of CD20 in DLBCL cell EVs has been demonstrated earlier (Martínez et al., 2025) and we found it more prominently expressed in U‐2 and S‐4 cell line EVs. In patient serum EVs, however, regardless of the permeabilization step used here, the expression of CD20 was undetectable within the four patient samples assessed here.

Another interesting marker, also associated to BCL pathogenesis, a transmembrane receptor CD44, was examined here. We noted that both cell line and serum EVs showed a weak or non‐detectable signal without permeabilization, indicating either low abundance or epitope masking (Nagel et al., 2010). Intriguingly, the luminal marker HSP70 was barely detected under non‐permeabilized conditions in cell line EVs and serum EVs, while permeabilization step enabled a strong detection in serum EVs (Figure 5H&I).

The WB results validate the presence of both surface and luminal EV markers in cell line and patient‐derived samples. The differential detection of markers before and after permeabilization emphasizes the importance of this step, especially when investigating clinical samples where more complex EV surface may affect obscure antigenic epitopes.

## 4 Discussion

EVs are emerging as promising biomarkers in hematologic malignancies, yet their clinical application in lymphomas remains limited by challenges in subtype‐specific characterization, and detection in complex fluids such as serum. In this study, we applied a multimodal EV characterization pipeline combining SEC isolation, TEM + iEM, immunoassays, WB and MS to study EV characteristics in parallel in the two main DLBCL subtypes ABC and GCB and a limited, proof‐of‐concept cohort of lymphoma patient‐derived EVs. Our findings demonstrate that EVs derived from DLBCL cell lines exhibit distinct morphological and molecular profiles. Notably, we observed that ABC type cells release smaller EVs, are significantly more abundant with CD81 and CD9 than the GCB type that displayed comparatively larger EVs and lower tetraspanin marker expression. These differences were consistent with MS, TRFIA and BSFIA immunoassays, WB and iEM analysis.

Interestingly, both CD81 and CD9 were preferentially enriched in larger EVs (>100 nm), suggesting that marker distribution may be linked not only to cell‐of‐origin but also by EV subpopulation. These findings extend previous reports of tetraspanin heterogeneity in cancer‐derived EVs and suggest that tetraspanins, and CD81 in particular, may serve as a one potential biomarker for stratifying DLBCL subtypes in future liquid biopsy applications (Casari et al., 2021; Islam et al., 2019). However, we did not study the patient‐derived material for the expression here and this finding on tetraspanin expression enrichment in ABC subtypes needs to be extended and confirmed in a cohort consisting of ABC, GCB and healthy donor serum or plasma samples.

In addition to cell line‐derived EVs, we investigated the EVs isolated from the serum of lymphoma patients to investigate the possibility to detect B cell originating markers in the complex biofluids and to compare the methodological features between the sample types. Compared to cell line samples, serum EVs from four different type of lymphoma diagnoses expectedly displayed heterogeneity in size and morphological appearance. We applied the same experimentation to evaluate the samples, and MS analysis revealed that several markers were identified both in cell line and serum EVs. Validation experiments with ELISA and WB, however, could not be directly applied to serum EVs but required further optimization with sample permeabilization. With the permeabilization step, the molecules detected in MS (and also some that were not) could be successfully detected in immunoassays and WB assays. This optimization did not facilitate marker detection in the iEM, though. Attempts to permeabilize (with Triton‐X‐100) EVs to expose internal epitopes compromised EV binding to grids, further highlighting the technical challenges of EV imaging in biofluids and the need for further optimization of EM‐assisted marker detection. Nevertheless, both MS and WB analysis confirmed the presence of key EV markers including CD9, CD81, and HSP70 in patient‐derived samples, underscoring the utility of combining orthogonal techniques for comprehensive EV characterization.

Beyond their diagnostic potential, the observed differences in EV morphology and marker composition may also reflect underlying biological variations in EV release pathways between DLBCL subtypes. ABC type DLBCL is associated with a more aggressive clinical course and increased metabolic activity, which may contribute to the elevated total EV release observed in these cell lines. Furthermore, the higher tetraspanin expression in ABC cell EVs and size‐based enrichment of CD81 and CD9 in larger EVs suggests possible differences in subcellular origin or release into EVs; however, this aspect was not studied in this project. Interestingly, even if marked differences were seen with relative tetraspanin expression in the EVs, all the DLBCL cell lines expressed comparable CD81, CD9 and CD63 within the cell population when observed with immunofluorescent staining. The ability to distinguish DLBCL subtypes through non‐invasive EV characterization opens potential avenues for disease monitoring, especially in cases where tissue biopsies are inaccessible or high‐risk.

Our findings also offer valuable insights into methodological considerations for EV research in hematologic malignancies. SEC‐based isolation provided sufficient EV purity and yield for downstream analysis from both cell culture and serum, as reflected in the high number of EV‐associated proteins identified via DIA‐MS and consistent tetraspanin detection across assays. Each characterization method exhibited distinct strengths and limitations. Notably, the automated magnetic enrichment of lipid particles, aligning the protocol from Wu et al. (2025), from serum was effective and could serve as a less laborious method for EV proteome analysis. For targeted validation of specific markers, it was noticed that even if MS did not detect e.g., the B cell marker CD19, the protein was present within all the EVs when analysed by WB. This is a common finding for some surface‐tethered markers especially (Tran et al., 2025). Immunogold TEM successfully distinguished marker expression in cell line EVs, yet was hindered in serum derived EVs likely due to protein corona, a recurring obstacle in biofluid‐derived EV analysis (Willms et al., 2018). These technical findings support recent calls for method standardization and the use of complementary approaches to validate EV content and origin (Welsh et al., 2024).

Intriguing findings related to DLBCL subtype‐specificity were found in the MS analysis of cell line EVs: one of them was the anti‐apoptotic regulator BCL‐2 which is reported to be linked to poor prognosis and to have high expression in ABC subtype of the DLBCL (Iqbal et al., 2006; Vogler et al., 2025) and our unpublished observations). The BCL‐2 was detected only in ABC EVs in our dataset (Supplementary File 1). Another intriguing protein hit was G‐protein Gna13, involved in germinal centre B cell migration. Gna13, when mutated in GCB cells, results in the loss of Gna13 function thus promoting lymphoma in vivo and impaired apoptosis (Becker et al., 2016; Muppidi et al., 2014);). Our MS analysis showed that the ABC cell line EVs were highly enriched with this regulator in comparison to GCB cell lines (Supplementary File 1), suggesting a differential expression between main DLBCL subtypes. Furthermore, a group of germinal centre‐linked immunoglobulin chains was represented exclusively in GCB derived cell line EV samples: IGHG1, IGHG2 and IGHG4, immunoglobulin gamma heavy chains ‐1, ‐2 and ‐4, reported specifically in germinal centre B cell class‐switching function. These molecules were either highly overexpressed or exclusively detected in GCB EV samples (Sundling et al., 2021; Supplementary File 1) demonstrating another subtype‐specific trait in DLBCL subtype ‐derived vesicles (Leveille & Johnson, 2021). Interestingly, markers related to heat shock responses (Hsp70, Hsp90) were more abundant in GCB type EVs (Supplementary File 1) but could not be successfully detected in cell line EVs in WB analysis as in the serum EVs. Of great interest are also the common regulators such as CD44 and Gal3BP found in high abundance in both lymphoma cell line and patient serum EVs (Supplementary Files 1–3) and these should be studied for their presence in a larger lymphoma panel together with matched samples from healthy donors. Furthermore, the B cell specific signature marker CD19 with varyingly sized isoform could be detected in the serum EVs, indicating a significant presence of B cell derived EVs that can be utilized for BCL targeted analytics.

In this study, certain limitations should be acknowledged. The patient cohort was small and not confined to DLBCL subtypes, and future studies involving larger, clinically stratified patient groups will be essential to draw any translational conclusions on marker expression or to validate the generalizability of these findings. Moreover, while our data reveal possibly subtype‐specific EV marker profiles, functional assays were not conducted to explore the biological roles of these vesicles in DLBCL progression or immune modulation. Future work will include a larger cell line panel and could additionally include co‐culture models or in vivo studies to investigate the role of subtype‐enriched EVs in tumour behaviour, immune evasion, or drug resistance. Finally, the application of more advanced EV separation methods (e.g., asymmetric flow field‐flow fractionation or immunocapture) and multi‐omic approaches could provide deeper insights into the molecular cargo of DLBCL‐derived EVs and their translational relevance. Lastly, the impurities in the SEC‐isolated EV population makes some conclusions difficult to make as a part of the effects in vesicle size determination for example may come from various types of particles e.g. apolipoproteins and albumin. Nevertheless, we believe that the population level analysis of a diverse vesicle entity is important in order to understand the holistic determinants and variability in the common sample types and their possible co‐function.

In conclusion, our study presents a comprehensive, multi‐platform analysis of EV population derived from DLBCL cell lines, revealing subtype‐dependent differences in EV size, and marker expression. By integrating orthogonal techniques, we provide evidence that tetraspanin markers, particularly CD9 and CD81, may differentiate DLBCL at the EV level, although they could also reflect cell source differences. Thus, future work with a more comprehensive DLBCL cell line catalogue and patient sample cohort with diagnostic insight into prior DLBCL subtyping would be needed to exclusively link the tetraspanin expression to a specific subtype. However, although limited dataset, our data support the feasibility of EV‐based approaches for disease stratification and lay the groundwork for future biomarker development in liquid DLBCL biopsy applications.

## 5 Conclusions

This study demonstrates that EVs isolated from DLBCL cell lines and patient serum carry distinct morphological and molecular signatures that may reflect the disease's molecular subtypes. Using a multimodal characterization pipeline, we identified reproducible enrichment of tetraspanin markers CD81 and CD9 in EVs derived from ABC type DLBCL cells compared to GCB type. An intriguing finding is also the possible dimerized presence of CD19 in serum EVs, indicative of successful and distinct lymphoma EV identification from the mixture of vesicles from various cell sources. These findings support the potential of EVs as non‐invasive biomarkers for B cell lymphomas and highlight the importance of using complementary techniques to overcome methodological challenges in different sample types. Future studies should explore a larger sample cohort and the functional relevance of subtype‐specific EVs and their applicability in disease stratification and monitoring.

## Author Contributions

**Nasir Badal**: conceptualization; data curation; formal analysis; investigation; methodology; visualization, project administration, writing – original draft, writing – review and editing. **Tiia Koivula**: data curation; formal analysis, resources, writing – review and editing. **Khirul Islam**: methodology, supervision, investigation; writing – review and editing. **Laura Lehtinen**: resources, review and editing. **Otto Kauko**: methodology, writing – review and editing. **Janne Leivo**: methodology, supervision, resources, review and editing. **Ilkka Heinonen**: supervision, resources, funding acquisition, review and editing. **Saara Hämälistö**: conceptualization, funding acquisition, supervision, methodology, project administration; resources; visualization; writing – original draft, writing – review and editing.

## Ethics Statement

All applicable international, national and/or institutional guidelines regarding the use of patient material were followed. The use of patient‐derived material was under license (ethical number 72/1801/2018) by Turku University Hospital (I.H.). The study follows the principles of the Declaration of Helsinki.

## Conflicts of Interest

The authors declare no conflicts of interest.

## Supporting information




**Supplementary Material**: jex270107‐sup‐0001‐SuppMat.xlsx

**Supplementary Material**: jex270107‐sup‐0002‐SuppMat.xlsx

**Supplementary Material**: jex270107‐sup‐0003‐SuppMat.xlsx

**Supplementary Material**: jex270107‐sup‐0004‐SuppMat.xlsx

**Supplementary Figure 1**. Analyzing the SEC fractions for tetraspanin expression enrichment in lymphoma culture supernatants and patient serum. The SEC fractions were collected as four fractions (F1‐F4) each containing 340 µL of flow‐through. (A‐B) TRFIA signal for the relative expression of tetraspanins CD81 (A) and CD63 (B) in SEC fractions (F1‐F4) from DLBCL cell lines supernatants (U‐2, Ri, S‐4, and O‐7). Tetraspanin levels were highest in fractions F1‐F3. The results shown are based on three biological replicates (n = 3). (C) Negative‐stain TEM images of U‐2 cell lines supernatants SEC fractions showed the highest presence of vesicle‐like structures in F1‐F2. In F3, EVs were still present but accompanied by additional soluble protein material, while F4 contained a large fraction of non‐EV protein material (scale bars, 200 nm). (D‐E) Western blot analysis of fractions from the four‐cell line supernatant show enrichment of CD9 and CD81 in F1‐F3, with reduced GAPDH and apolipoprotein ApoA1 contamination. Red boxes highlight the first three fractions enriched with tetraspanins in the Western blots that were used in down‐stream analyses. Whole cell lysates (UCL for U‐2, RCL for Ri, SCL for S‐4, OCL for O‐7) serve as marker expression controls. (F‐G) Western blots of SEC fractions from serum samples of four lymphoma patients (Pt#01–Pt#04), display the most enriched CD9/CD81 expression in F1‐F3 fractions.
**Supplementary Figure 2**. Immunoassay and immunostaining confirm tetraspanin marker expression. (A) Quantification of CD63 expression on cell line‐derived EVs using europium nanoparticle‐based assays. Y‐axis in panel A is shown on a log10 scale. (B) CD9 expression in serum‐derived EVs from four BCL patients (Pt#01–Pt#04), compared to IgG1 isotype and plain serum controls. Data represent the mean from three independent experiments. C) The U‐2, Ri, S‐4 and O‐7 cells were allowed to adhere on a‐IgM/IgG‐coated coverslips for 45 minutes, fixed, permeabilized and stained with a‐CD63 (grey colour, upper panel), a‐CD81 (magenta, upper panel) or a‐CD9 (grey colour, lower panel) antibodies and DAPI (blue colour) for nuclear stain and imaged with spinning‐disc confocal microscope. Maximum projections of images are shown, scale bars 10 µm.
**Supplementary Figure 3**. Size distribution immunogold TEM analysis of CD81 expression in EVs from DLBCL cell lines. (A) Representative TEM image of CD81‐labeled EVs from U‐2932 (ABC subtype) showing gold particle localization (red arrows). EVs larger than 100 nm are marked in magenta; EVs smaller than 100 nm are marked in cyan. (B) Average percentage of CD81‐positive EVs across all size ranges in four DLBCL cell lines. (C, D) Proportion of CD81‐positive EVs in the <100 nm (C) and >100 nm (D) size categories, showing higher positivity in larger EVs from ABC subtypes. (E, F) Total EV counts analysed in each size category for small (<100 nm; E) and large (>100 nm; F) vesicles. Overall, ABC‐type EVs (U‐2932, Riva) displayed significantly greater CD81 surface labelling than GCB‐type EVs (SUDHL‐4, OCI‐LY7), particularly within the >100 nm population. Data represent means from three independent experiments (total n = 300; n = 100 per experiment). Scale bar = 100 nm.
**Supplementary figure 4**. Proteomic profiling of patient serum‐derived (EVs). (A) Heatmap displaying the normalized abundance of selected common EV‐associated proteins identified via MS in SEC‐isolated EVs from DLBCL cell lines (U‐2, Ri, S‐4, & O‐7) and patient serum samples (Pt#01‐Pt#04). Missing values are indicated by white boxes.
**Supplementary figure 5**. WB validation of EV‐associated markers in DLBCL‐derived EVs. (A) Sample processing steps prior to WB (B, C) WB validation of selected surface markers (CD44, CD81) in SEC‐isolated EVs under non‐permeabilized and permeabilized conditions. Ponceau S staining shows the presence of protein bands both in permeabilized and non‐permeabilized blots. Cell lysates and ladder were included as controls. *Created in BioRender. Hämälistö, S. (2026)* https://BioRender.com/c0sjwwo.

## Data Availability

All raw data analysed in this study is available upon request.
