# Supplementary material for: Molecular Characterization of Extracellular Vesicles From Human B Cell Lymphomas: Methodological Comparison to Vesicles From Patient Serum
Source: J Extracell Biol. 2026 Feb 22;5(2):e70107. doi: 10.1002/jex2.70107 (PMC12927977; doi:10.1002/jex2.70107)
Supplement: Supplementary file 5 — Supplementary Figure 1. Analyzing the SEC fractions for tetraspanin expression enrichment in lymphoma culture supernatants and patient serum. The SEC fractions were collected as four fractions (F1‐F4) each containing 340 µL of flow‐through. (A‐B) TRFIA signal for the relative expression of tetraspanins CD81 (A) and CD63 (B) in SEC fractions (F1‐F4) from DLBCL cell lines supernatants (U‐2, Ri, S‐4, and O‐7). Tetraspanin levels were highest in fractions F1‐F3. The results shown are based on three biological replicates (n = 3). (C) Negative‐stain TEM images of U‐2 cell lines supernatants SEC fractions showed the highest presence of vesicle‐like structures in F1‐F2. In F3, EVs were still present but accompanied by additional soluble protein material, while F4 contained a large fraction of non‐EV protein material (scale bars, 200 nm). (D‐E) Western blot analysis of fractions from the four‐cell line supernatant show enrichment of CD9 and CD81 in F1‐F3, with reduced GAPDH and apolipoprotein ApoA1 contamination. Red boxes highlight the first three fractions enriched with tetraspanins in the Western blots that were used in down‐stream analyses. Whole cell lysates (UCL for U‐2, RCL for Ri, SCL for S‐4, OCL for O‐7) serve as marker expression controls. (F‐G) Western blots of SEC fractions from serum samples of four lymphoma patients (Pt#01–Pt#04), display the most enriched CD9/CD81 expression in F1‐F3 fractions. Supplementary Figure 2. Immunoassay and immunostaining confirm tetraspanin marker expression. (A) Quantification of CD63 expression on cell line‐derived EVs using europium nanoparticle‐based assays. Y‐axis in panel A is shown on a log10 scale. (B) CD9 expression in serum‐derived EVs from four BCL patients (Pt#01–Pt#04), compared to IgG1 isotype and plain serum controls. Data represent the mean from three independent experiments. C) The U‐2, Ri, S‐4 and O‐7 cells were allowed to adhere on a‐IgM/IgG‐coated coverslips for 45 minutes, fixed, permeabilized and stained with [file JEX2-5-e70107-s004.docx]

**SUPPLEMENTARY FIGURES**

**Supplementary Figure 1.**


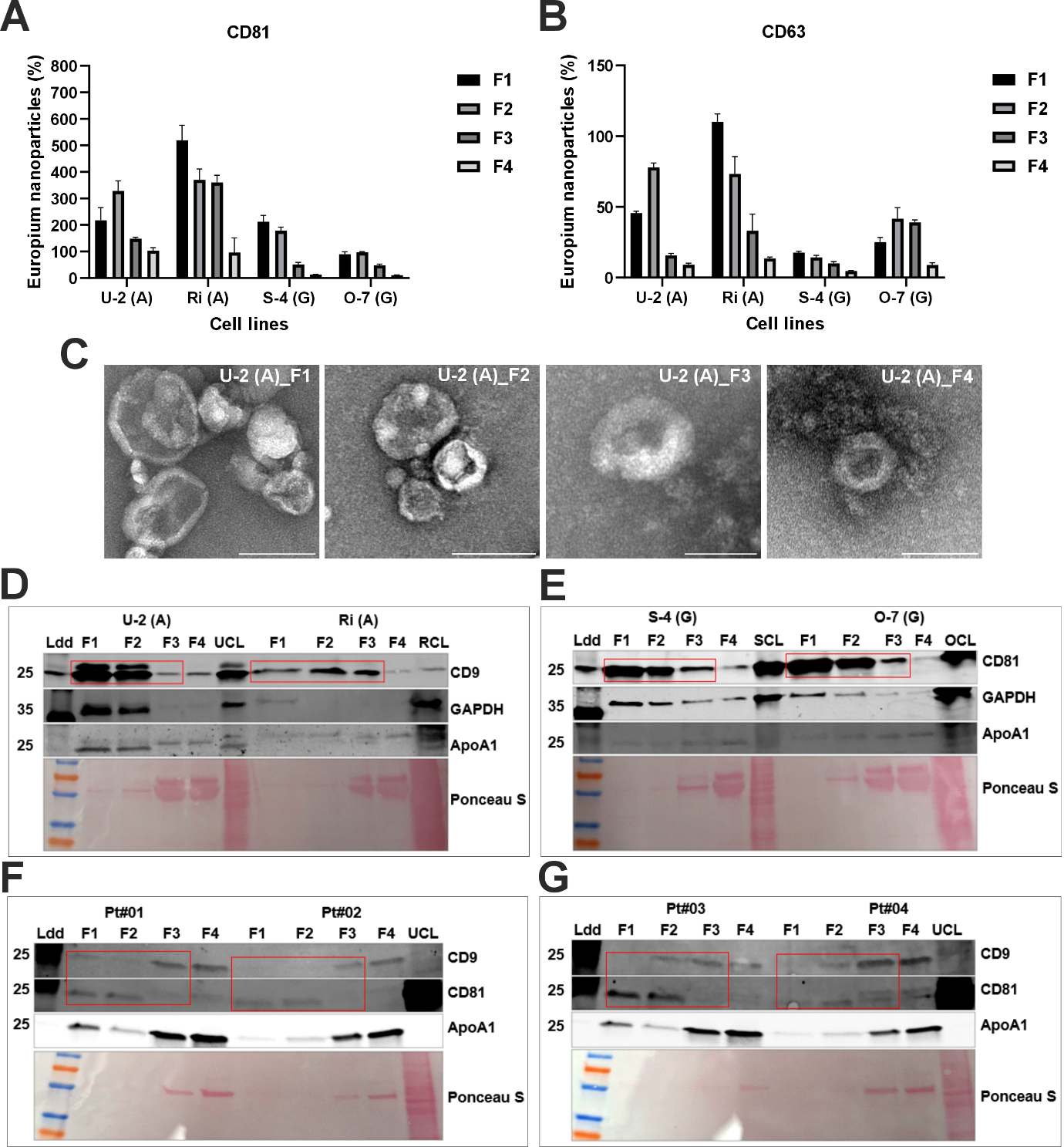


**Supplementary Figure 2.**


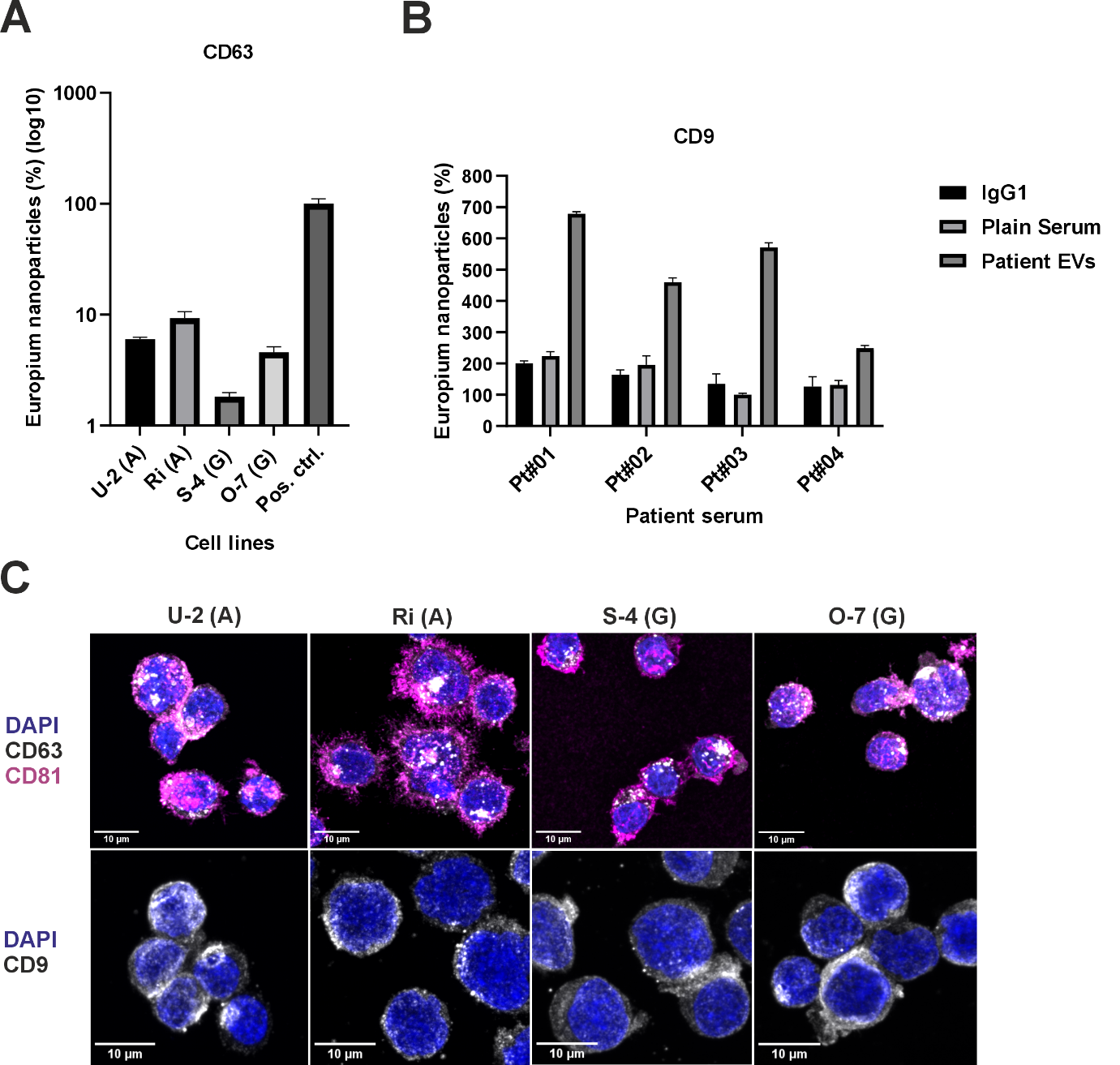


**Supplementary Figure 3.**


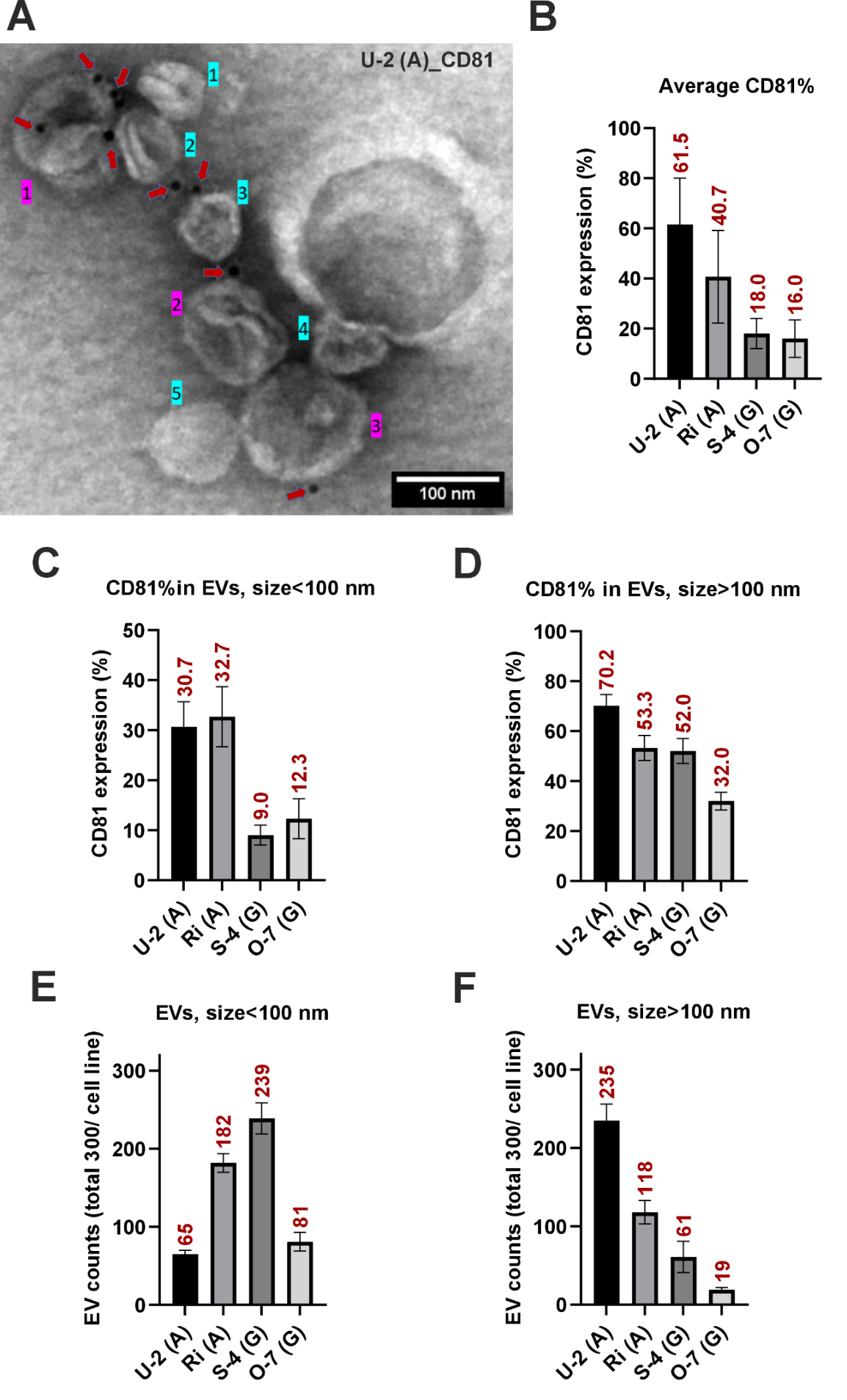


**Supplementary Figure 4.**


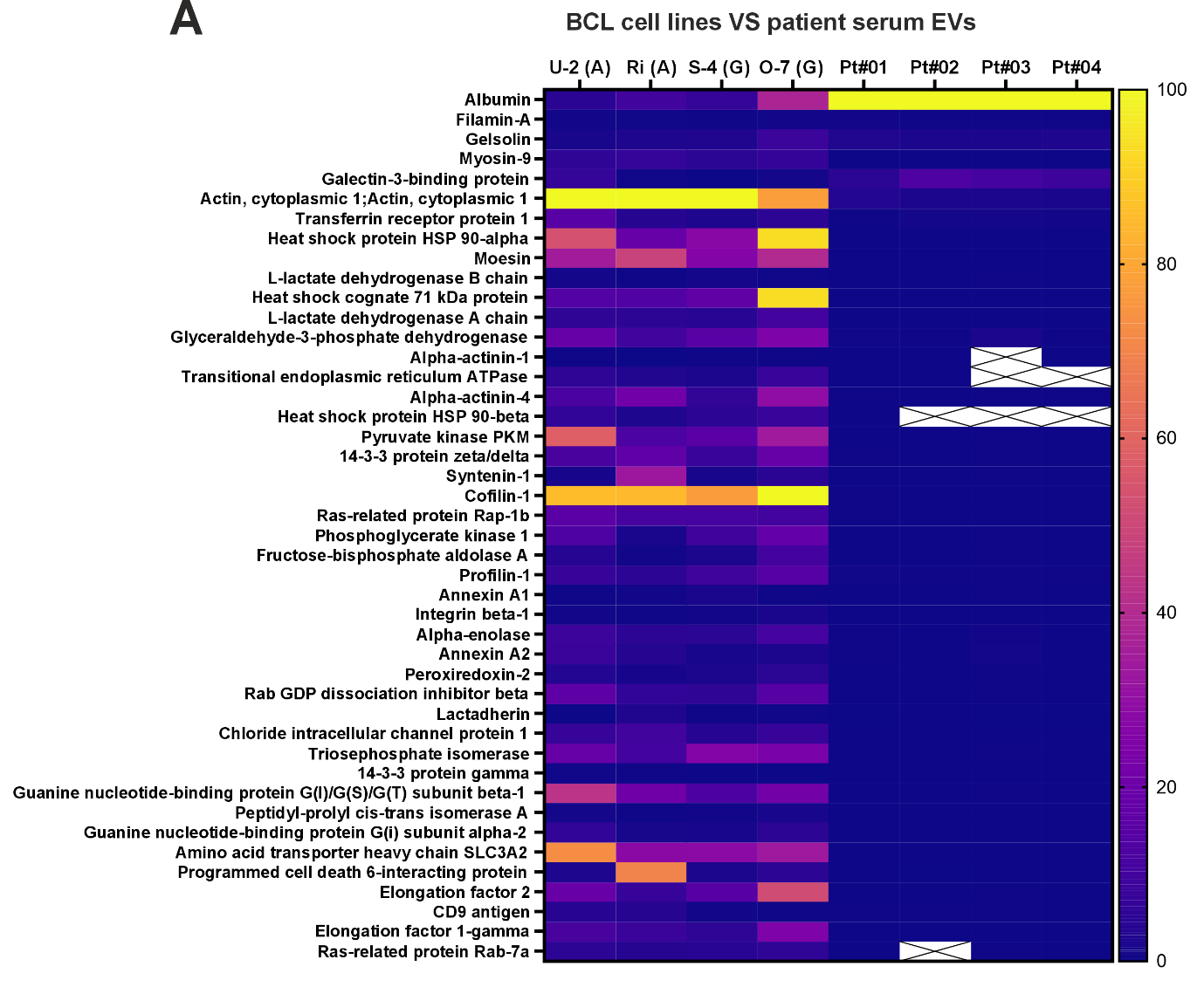


**Supplementary Figure 5.**


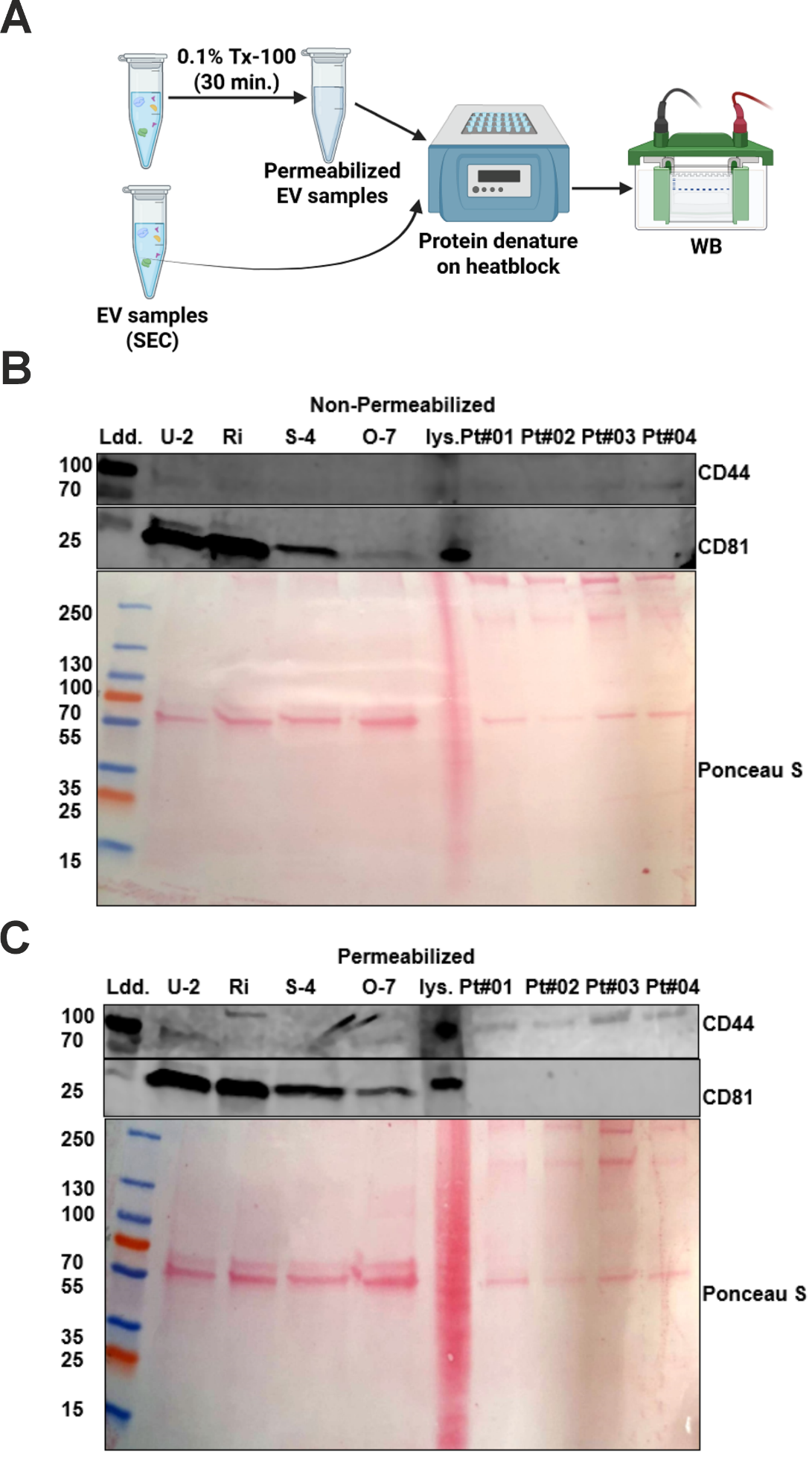


**SUPPLEMENTARY FIGURE CAPTIONS**

**Supplementary Figure 1. Analyzing the SEC fractions for tetraspanin expression enrichment in lymphoma culture supernatants and patient serum.** The SEC fractions were collected as four fractions (F1-F4) each containing 340 µl of flow-through. (A-B) TRFIA signal for the relative expression of tetraspanins CD81 (A) and CD63 (B) in SEC fractions (F1-F4) from DLBCL cell lines supernatants (U-2, Ri, S-4, and O-7). Tetraspanin levels were highest in fractions F1-F3. The results shown are based on three biological replicates (n=3). (C) Negative-stain TEM images of U-2 cell lines supernatants SEC fractions showed the highest presence of vesicle-like structures in F1-F2. In F3, EVs were still present but accompanied by additional soluble protein material, while F4 contained a large fraction of non-EV protein material (scale bars, 200 nm). (D-E) Western blot analysis of fractions from the four-cell line supernatant show enrichment of CD9 and CD81 in F1-F3, with reduced GAPDH and apolipoprotein ApoA1 contamination. Red boxes highlight the first three fractions enriched with tetraspanins in the Western blots that were used in down-stream analyses. Whole cell lysates (UCL for U-2, RCL for Ri, SCL for S-4, OCL for O-7) serve as marker expression controls. (F-G) Western blots of SEC fractions from serum samples of four lymphoma patients (Pt#01–Pt#04), display the most enriched CD9/CD81 expression in F1-F3 fractions.

**Supplementary Figure 2. Immunoassay and immunostaining confirm tetraspanin marker expression.** (A) Quantification of CD63 expression on cell line-derived EVs using europium nanoparticle-based assays. Y-axis in panel A is shown on a log10 scale. (B) CD9 expression in serum-derived EVs from four BCL patients (Pt#01–Pt#04), compared to IgG1 isotype and plain serum controls. Data represent the mean from three independent experiments. C) The U-2, Ri, S-4 and O-7 cells were allowed to adhere on a-IgM/IgG-coated coverslips for 45 minutes, fixed, permeabilized and stained with a-CD63 (grey color, upper panel), a-CD81 (magenta, upper panel) or a-CD9 (grey color, lower panel) antibodies and DAPI (blue color) for nuclear stain and imaged with spinning-disc confocal microscope. Maximum projections of images are shown, scale bars 10 μm.

**Supplementary Figure 3. Size distribution immunogold TEM analysis of CD81 expression in EVs from DLBCL cell lines.** (A) Representative TEM image of CD81-labeled EVs from U-2932 (ABC subtype) showing gold particle localization (red arrows). EVs larger than 100 nm are marked in magenta; EVs smaller than 100 nm are marked in cyan. (B) Average percentage of CD81-positive EVs across all size ranges in four DLBCL cell lines. (C, D) Proportion of CD81-positive EVs in the <100 nm (C) and >100 nm (D) size categories, showing higher positivity in larger EVs from ABC subtypes. (E, F) Total EV counts analyzed in each size category for small (<100 nm; E) and large (>100 nm; F) vesicles. Overall, ABC-type EVs (U-2932, Riva) displayed significantly greater CD81 surface labeling than GCB-type EVs (SUDHL-4, OCI-LY7), particularly within the >100 nm population. Data represent means from three independent experiments (total n=300; n=100 per experiment). Scale bar = 100 nm.

**Supplementary figure 4. Proteomic profiling of patient serum-derived (EVs).** **(A)** Heatmap displaying the normalized abundance of selected common EV-associated proteins identified via mass spectrometry in SEC-isolated EVs from DLBCL cell lines (U-2, Ri, S-4, & O-7) and patient serum samples (Pt#01-Pt#04). Missing values are indicated by white boxes

**Supplementary figure 5. WB validation of EV-associated markers in DLBCL-derived EVs.** (A) Sample processing steps prior to WB (B, C) WB validation of selected surface markers (CD44, CD81) in SEC-isolated EVs under non-permeabilized and permeabilized conditions. Ponceau S staining shows the presence of protein bands both in permeabilized and non-permeabilized blots. Cell lysates and ladder were included as controls.
